# Spectroscopic Identification of Active Sites of Oxygen‐Doped Carbon for Selective Oxygen Reduction to Hydrogen Peroxide

**DOI:** 10.1002/anie.202303525

**Published:** 2023-04-18

**Authors:** Longxiang Liu, Liqun Kang, Arunabhiram Chutia, Jianrui Feng, Martyna Michalska, Pilar Ferrer, David C. Grinter, Georg Held, Yeshu Tan, Fangjia Zhao, Fei Guo, David G. Hopkinson, Christopher S. Allen, Yanbei Hou, Junwen Gu, Ioannis Papakonstantinou, Paul R. Shearing, Dan J. L. Brett, Ivan P. Parkin, Guanjie He

**Affiliations:** ^1^ Christopher Ingold Laboratory Department of Chemistry University College London 20 Gordon Street London WC1H 0AJ UK; ^2^ Department of Inorganic Spectroscopy Max-Planck-Institute for Chemical Energy Conversion Stiftstr. 34–36 45470 Mülheim an der Ruhr Germany; ^3^ School of Chemistry University of Lincoln Lincolnshire LN6 7DL UK; ^4^ Photonic Innovations Lab Department of Electronic & Electrical Engineering University College London Torrington Place London WC1E 7JE UK; ^5^ Diamond Light Source Rutherford Appleton Laboratory Harwell, Didcot OX11 0DE UK; ^6^ electron Physical Science Imaging Centre Rutherford Appleton Laboratory Harwell, Didcot OX11 0DE UK; ^7^ Department of Materials University of Oxford Parks Road Oxford OX1 3PH UK; ^8^ HP-NTU Digital Manufacturing Corporate Laboratory School of Mechanical and Aerospace Nanyang Technological University 50 Nanyang Avenue Singapore 639798 Singapore; ^9^ Electrochemical Innovation Lab Department of Chemical Engineering University College London London WC1E 7JE UK

**Keywords:** Electrocatalysis, Hydrogen Peroxide, NEXAFS, Porous Carbon, Quinone

## Abstract

The electrochemical synthesis of hydrogen peroxide (H_2_O_2_) via a two‐electron (2 e^−^) oxygen reduction reaction (ORR) process provides a promising alternative to replace the energy‐intensive anthraquinone process. Herein, we develop a facile template‐protected strategy to synthesize a highly active quinone‐rich porous carbon catalyst for H_2_O_2_ electrochemical production. The optimized PCC_900_ material exhibits remarkable activity and selectivity, of which the onset potential reaches 0.83 V vs. reversible hydrogen electrode in 0.1 M KOH and the H_2_O_2_ selectivity is over 95 % in a wide potential range. Comprehensive synchrotron‐based near‐edge X‐ray absorption fine structure (NEXAFS) spectroscopy combined with electrocatalytic characterizations reveals the positive correlation between quinone content and 2 e^−^ ORR performance. The effectiveness of chair‐form quinone groups as the most efficient active sites is highlighted by the molecule‐mimic strategy and theoretical analysis.

## Graphical abstract

The quinone‐doped carbon electrocatalyst exhibits a remarkable onset potential of 0.83 eV vs. RHE in 0.1 M KOH and over 95 % H_2_O_2_ selectivity in both alkaline and neutral electrolytes. The effectiveness of chair‐form quinone groups as the most efficient active sites is highlighted by the reverse verification method, compared with other oxygen functional groups.

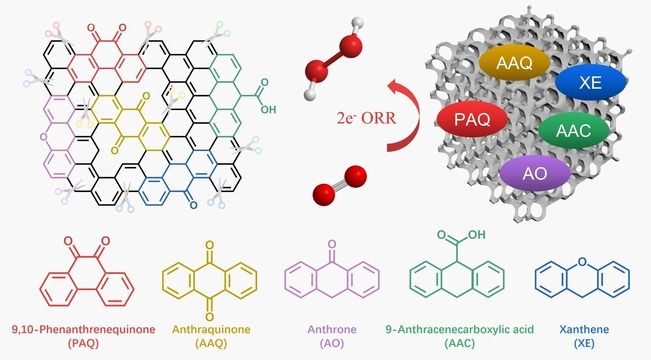

## Introduction

Since its discovery by Louis Jacques Thénard in 1818, hydrogen peroxide (H_2_O_2_) has been extensively applied in diverse fields such as environmental remediation, pharmaceutical synthesis, and paper bleaching, owing to its strong oxidizing ability.^[1]^ Especially since the outbreak of the COVID‐19 pandemic, the demand for hydrogen peroxide has grown rapidly due to its prevalence in disinfectant products.^[2]^ The ever‐growing demand for H_2_O_2_ has led to the search for more efficient and inexpensive production methods in the industry. In contrast to the traditional energy‐intensive anthraquinone process and high‐cost direct heterogeneous synthesis from gaseous H_2_ and O_2_ which requires high temperature and pressurized reactors, electrocatalytic H_2_O_2_ production via a two‐electron (2 e^−^) pathway of oxygen reduction reaction (ORR) provides a straightforward and environmentally‐friendly alternative for the on‐site production of H_2_O_2_ under mild conditions, and, overall, greatly reduces the cost and risk of product transportation and storage.^[3]^

The feasibility of the implementation of electrocatalytic H_2_O_2_ production is highly dependent on the development of electrocatalysts, which requires not only high electrocatalytic activity and H_2_O_2_ selectivity, but also long‐lasting durability and economic viability. Up to now, numerous electrocatalysts have been developed, including noble metals and their alloys,^[4]^ transition metal sulfides,^[5]^ metal‐doped carbon materials,^[6]^ and metal‐free carbon materials.^[7]^ Of these, metal‐free carbon materials have attracted considerable attention due to their high selectivity, stability, electrical conductivity, and low cost, as well as adaptable structures and surfaces.^[8]^ In particular, oxygen functional groups (OFGs) have been identified as the most significant components for improving the selectivity of H_2_O_2_.^[3]^ Various fabrication approaches have been proposed to introduce OFGs onto carbon surfaces. However, complicated synthesis routes and techniques, involving harsh oxidation conditions and complex mechanochemical treatments, are commonly required, thus hampering their further practical applications.^[9]^ Additionally, the introduction of OFGs is usually restricted to the limited surface of the catalysts, reducing the volumetric efficiency. For example, although high H_2_O_2_ selectivity (over 90 %) is realized on nitric acid‐oxidized CNT (O‐CNT) material, its activity is limited by the low electrochemically active surface area, of which the onset potential is only 0.75 V vs. reversible hydrogen electrode (RHE) to reach the current density of 0.1 mA cm^−2^.^[9a]^

On the other hand, the identification of active OFGs remains a considerable challenge due to the lack of an in‐depth understanding of the underlying mechanism, precise synthesis strategies, and exhaustive characterization methods. To prove the role of each OFG, chemical titration has been identified as a good strategy by selectively blocking some specific OFGs.^[10]^ However, the incorporation of titrant groups would inevitably deteriorate 2 e^−^ ORR performance, thus complicating the identification of OFGs. For example, Chen et al. employed a chemical titration strategy to selectively eliminate partial OFGs on the carbon surface and proposed that C=O groups are the most active sites compared to C−OH and COOH groups.^[11]^ However, employing the same method, Lim et al. proposed that COOH groups are more active than C=O and C−OH groups.^[12]^ Therefore, facile and efficient strategies are required to explore the active sites of carbon catalysts and elucidate the active sites for the electrocatalytic production of H_2_O_2_. To identify different OFGs, near‐edge X‐ray absorption fine structure (NEXAFS) spectroscopy analysis has been increasingly employed because of its unique sensitivity in identifying electronic bonding/orbitals.^[13]^ However, due to the inevitable presence of oxygen species (e.g., from surface contamination), additional care must be taken into sample preparation and measurements to eliminate such interference. Another critical issue is that the inappropriate NEXAFS analysis even leads to misleading conclusions.

Herein, we propose a simple silica template‐protected strategy to prepare efficient porous carbon catalysts (PCCs) for electrocatalytic production of H_2_O_2_ through a 2 e^−^ ORR process. Due to the inhibition effect of tannic acid (TA) decomposition provided by the silica template during the annealing process, rich quinone groups are conserved after etching treatment. We show that quinone groups implanted PCCs exhibit excellent activity and selectivity for electrocatalytic production of H_2_O_2_. The high activity and selectivity (>95 %) within a large potential range in both alkaline and neutral electrolytes surpass not only most state‐of‐the‐art carbon catalysts but even most metal‐based catalysts, showing great potential for industrial applications in a wide pH condition. The presence of quinone groups is qualified and quantified by NEXAFS analysis and it is found to be positively correlated with 2 e^−^ ORR activity and selectivity. We also find that sample preparation methods are crucial in reducing contaminant oxygen species from the substrate, which can guide future NEXAFS users for catalysis and materials research. A molecule‐mimic strategy is applied to build aromatic molecules with individual OFGs on the H_2_‐reduced PCC_900_ through a simple solvothermal method to reveal the role of each OFG, where quinone, ketone, and carboxylic acid groups contribute to the 2 e^−^ ORR process, whereas the ether group tends to undergo a 4 e^−^ ORR process in the neutral electrolyte. Moreover, the 9,10‐phenanthrenequinone‐decorated material presents the best 2 e^−^ ORR activity and selectivity, further verifying the excellent performance of quinone groups compared to ketone and carboxylic acid groups. Finally, density functional theory calculations are employed to reveal the superiority of chair‐form quinone groups.

## Results and Discussion

PCCs are prepared through a typical sacrificial template method, as illustrated in Figure S1a. Homogeneous mixtures of TA and SiO_2_ nanospheres are prepared and subsequently carbonized at 900 °C in an Ar atmosphere. Afterwards, the SiO_2_ template is removed by alkaline etching treatment. Scanning electron microscopy (SEM), high‐angle annular dark‐field scanning transmission electron microscopy (HAADF‐STEM), and bright‐field scanning transmission electron microscopy (BF‐STEM) images of PCC_900_ in Figure S1b–d, reveal the distinct three‐dimensional amorphous porous structures with abundant nano‐ (<2 nm) and meso‐pores (2–50 nm).

The N_2_ adsorption‐desorption analysis is carried out to determine the porous properties of PCC_900_ (Figure S2), confirming a characteristic mesoporous structure with a high Barrett–Emmett–Teller (BET) surface area of 1466.92 m^2^ g^−1^ and a specific pore volume of 3.67 cm^3^ g^−1^. Due to the high pore volume and high BET surface, rich active sites are exposed, and the mass transfer of reactants is greatly improved. The morphologies and porous properties of PCCs with different mass ratios of TA to SiO_2_ nanospheres are evaluated in Figure S2–3, and Table S1. A ratio of 5 : 9 of TA to SiO_2_ nanospheres is employed to perform electrochemical characterization, as the largest BET surface area is achieved at this ratio.

Interestingly, besides its function as a template, we find that the SiO_2_ template also improves the thermal stability of the TA precursor. The thermal stability of pure TA and TA@SiO_2_ composites is evaluated by thermal gravimetric analysis (TGA) (Figure S4). After removing the SiO_2_ template, the TA−SiO_2_ composite exhibits a higher initial decomposition temperature (20 % of weight loss) which is 74 °C higher than that of pure TA (344 °C vs 270 °C). Moreover, the maximum derivative weight loss is postponed to 480 °C from 274 °C in the presence of SiO_2_ template, and the thermal stability at the high range of 400–800 °C is greatly elevated. We assume that SiO_2_ template acts as a physical isolating layer that inhibits the release of volatile species, thus improving its thermal stability, which is also evidenced by the SEM and digital images of annealed TA and TA−SiO_2_ materials in Figure S5. The annealed TA material forms a char foam due to the release of volatile species consisting of irregular carbon cracks with a size of tens of micrometers, whereas a dense char powder is obtained after annealing TA@SiO_2_ material due to the protective role of SiO_2_ nanospheres. More importantly, a large number of quinone groups, which are more stable than other OFGs, are formed and remain due to the template‐protection effect.^[14]^ They are proved to be responsible for high‐performance electrocatalytic H_2_O_2_ production in the following experiments.

**Figure 1:**
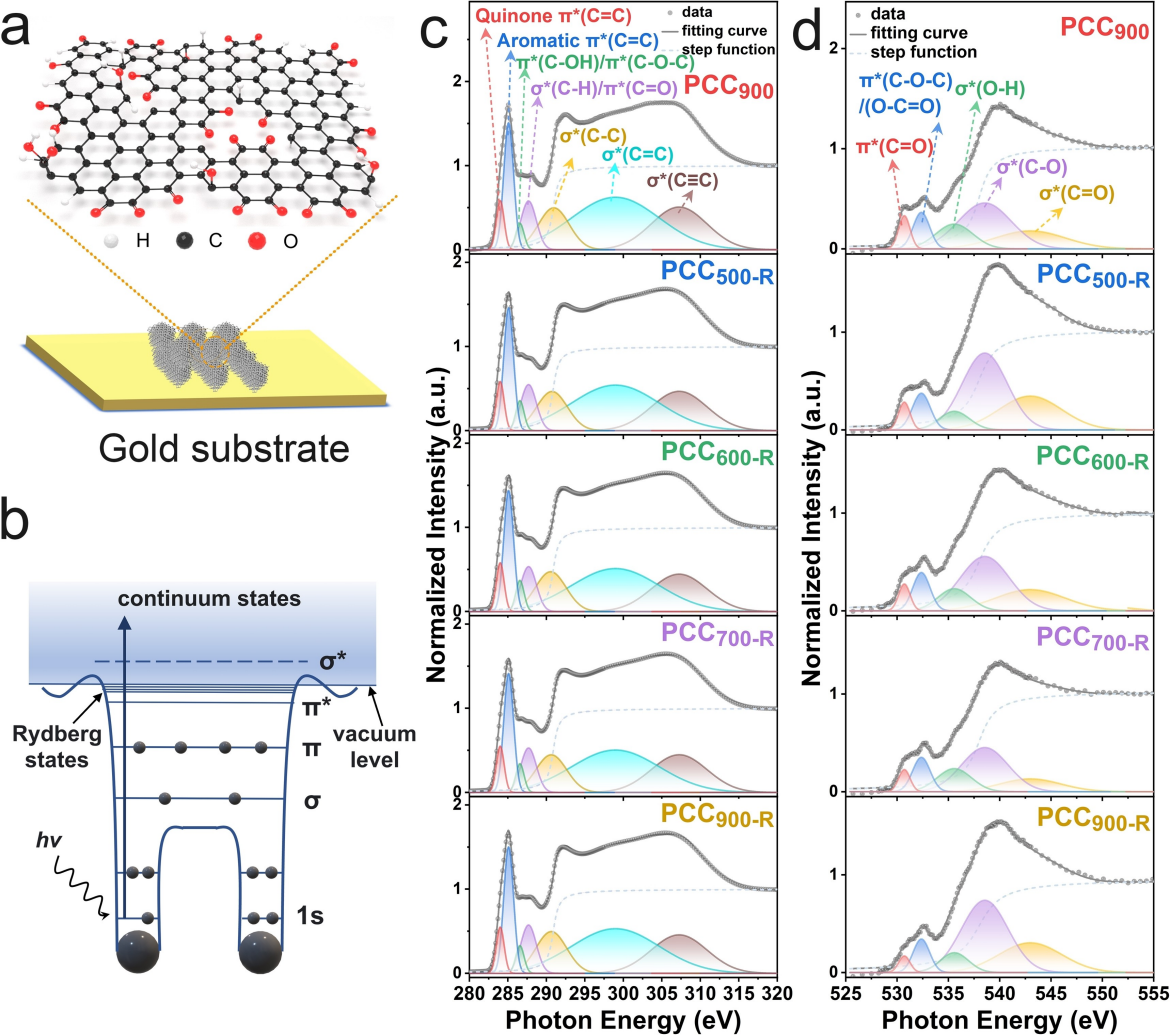
a) Schematic illustration of PCC_900_ on a gold substrate. b) Schematic illustration of an X‐ray adsorption process of a diatomic molecule. c) C K‐edge NEXAFS spectra measured on an indium substrate. d) O K‐edge NEXAFS spectra measured on a gold substrate.

As quinone groups are expected to decompose above 600 °C,[^14^, ^15^] the PCC_900_ material is re‐annealed at 500 °C, 600 °C, 700 °C, 900 °C in an Ar atmosphere (denoted as PCC_500‐R_, PCC_600‐R_, PCC_700‐R_, and PCC_900‐R_, respectively) to prepare catalysts with different oxygen contents. TGA measurements demonstrate that PCC_900_ and PCC_500‐R_ show clear weight loss from 600 °C due to the decomposition of OFGs (Figure S6). X‐ray photoelectron spectroscopy (XPS) is conducted to study their surface chemical properties (Figure S7 and S8). The oxygen content decreases from 7.9  at. % to 5.3 at. % when PCC_900_ is re‐annealed at 500 °C for 1 h, and it further decreases to 4.5 at. % for PCC_900‐R_, indicating that the OFGs are partly removed without protection from SiO_2_ templates. The content of the C=O group in the deconvoluted C 1s spectra decreases from 7.9 % to 5.8 % for PCC_900_ and PCC_900‐R_, whereas it is 7.5 % for PCC_500‐R_, indicating that the C=O group does not show a significant loss at 500 °C. In the deconvoluted O 1s spectra, the content of the C=O group of PCC_500‐R_ is higher than that of PCC_900_, due to the removal of OH or COOH groups.^[9a]^ When the re‐annealing temperature is increased to 900 °C, the proportion of C=O groups is reduced to 35.0 %, indicating the loss of C=O or quinone groups. Nevertheless, due to the shortcoming of XPS analysis, such as the proximity of binding energies for different OFGs and insufficient vacuum condition to rule out the effect of residual air, accurate identification of the variations in different OFGs requires more advanced techniques, such as NEXAFS.^[16]^ Meanwhile, both the transmission and attenuated total reflectance (ATR) Fourier‐transform infrared spectra (FTIR) fail to reveal the difference for the series of re‐annealed materials due to the strong adsorption of carbon background (Figure S9). Figure 1b displays a simplified schematic illustration of an X‐ray adsorption process of a diatomic molecule. The information of unoccupied orbitals is provided through interpreting the spectroscopic fingerprints caused by the excited electron transition from the 1 s core level to unoccupied 2p valence orbitals. Due to the high sensitivity of NEXAFS, subtitle change in electronic structures can be measured, which is not feasible for XPS analysis.^[13]^ Furthermore, the standard NEXAFS measurement and analysis is of primary significance to reveal the accurate electronic structures, which, however, is mostly missing in previous works and leads to ambiguous or even misleading results.[^9b^, ^11^]

**Figure 2:**
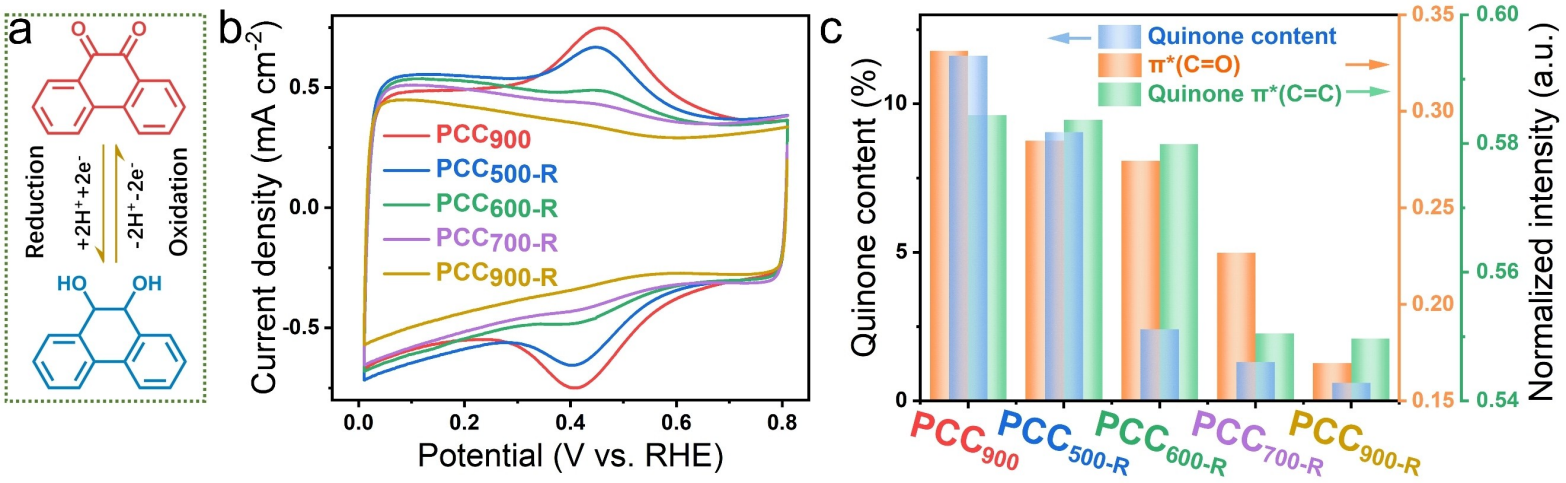
a) Schematic illustration of the quinone redox reaction process. b) CV curves measured in N_2_‐saturated 0.1 M PBS with a scan rate of 50 mV s^−1^. c) Comparison of quinone π* (C=C) and π* (C=O) intensity in NEXAFS spectra and quinone content obtained from CV results.

NEXAFS measurements are performed to investigate the precise electronic bonding of OFGs on PCCs in total electron yield (TEY) mode. Very comprehensive measurement and analysis information is provided in the materials characterization and supporting notes, which also provides clear guidance for future NEXAFS users. Indium plate, one of the widely used powder mounting substrates, is first used to load samples and the obtained C K‐edge NEXAFS spectra are displayed in Figure 1c and Figure S11. The characteristic transitions to unoccupied π* (C=C) and σ* (C=C) states are observed at 285.1 eV and 299.0 eV, respectively, for all prepared materials.^[17]^ This indicates that the aromatic hexagonal structure is mainly formed after the annealing process for PCC_900_. The peak at 286.6 eV is assigned to π* (C−OH) or π* (C−O−C) from the phenolic or etheric ring contributions.[^17^, ^18^] The peak at 287.7 eV is ascribed to the overlap of σ* (C−H) and π* (C=O).^[19]^ The broad peak at 291.0 eV and 307.2 eV are assigned to σ* (C−C) and σ* (C≡C), respectively.^[20]^ The peak at 284.0 eV can be interpreted as quinone π* (C=C) because of quinoid distortion.^[21]^ Compared with PCC_900_ and PCC_500‐R_, the intensity of quinone π* (C=C) decreases when PCC_900_ is re‐annealed at 600 °C, 700 °C, and 900 °C due to the partial decomposition of the quinone groups (Figure 2c, Figure S11b, and Supporting Note 1), which is in good accordance with the literature.^[14]^ PCC_900_ and re‐annealed materials exhibit a negligible difference in the C K‐edge NEXAFS spectra, revealing their similar carbon structures. Moreover, the bare indium plate shows a different spectrum in the range of 280–320 eV, indicating that the C K‐edge NEXAFS spectra of the samples are not contaminated by the indium plate where the samples are mounted, even when it appears to not be fully shielded by the sample (Figure S12b). However, the strong interference of the indium plate is observed in the O K‐edge spectra (Figure S12d). The peaks at around 532.6 eV, 535.4 eV, and 538.9 eV show overlaps with the signal of indium oxide, making it difficult to distinguish the specific oxygen electronic structure. Nevertheless, there is no peak from indium plate present at around 530.7 eV, which is ascribed to π* (C=O), originating from the quinone structure.^[22]^ The intensity of π* (C=O) decreases successively with increasing re‐annealing temperature from 500 °C to 900 °C (Figure S12c), further affirming the partial decomposition of the quinone groups.

To avoid the interference of the substrate in the oxygen spectra, the samples are deposited on clean gold‐coated silicon wafers (Supporting Note 3), and the obtained O K‐edge NEXAFS spectra are shown in Figure 1d. The bare gold substrate displays a significantly different spectrum in contrast to those with loaded samples (Figure S13a), demonstrating the suitability of gold substrates for O K‐edge NEXAFS measurement. The peak at 530.7 eV is assigned to transitions to unoccupied π* (C=O), originating from quinone groups.^[22]^ The normalized intensity of π* (C=O) decreases successively with increasing the re‐annealing temperature, further affirming the partial decomposition of the quinone groups (Figure S13b and Figure 2c). The actual difference in the normalized intensity of π* (C=O) of PCC_900_ and re‐annealed samples is expected to be more significant considering different amounts of oxygen atoms on the surface (Supporting Note 4). The ascription of the peak at 532.3 eV is complicated, and we tentatively assign it to a combination of π* (C−O−C)/(O−C=O) or π* (C−OH) due to the contributions from epoxide, ketone, or carboxyl groups.[^18^, ^22^, ^23^] The peak at 535.9 eV is assigned to σ* (O−H) from phenols or absorbed hydroxyl groups.^[23]^ The peaks at 539.2 eV and 543.1 eV are assigned to σ* (C−O) and σ* (C=O), respectively.^[17]^

Quinone structures are redox‐active, making them highly suitable for investigation via cyclic voltammetry (CV) (Figure 2a). As displayed in Figure 2b, characteristic redox peaks appear at around 0.4 V vs. RHE. The redox peak intensity decreases successively with increasing re‐annealing temperature, which is in good accordance with the NEXAFS analysis. The quinone content is calculated based on the integral ratio of peak areas (Figure S16) and i.e., 11.6 %, 9.1 %, 2.4 %, 1.3 %, 0.6 % for PCC_900_, PCC_500‐R_, PCC_600‐R_, PCC_700‐R_, PCC_900‐R_, respectively. Moreover, similar phenomena could be observed in N_2_‐saturated 0.1 M KOH (Figure S17). Therefore, based on the carbon and oxygen K‐edge NEXAFS analyses and CV measurements, it can be concluded that the quinone content is successively reduced for PCC_900_, PCC_500‐R_, PCC_600‐R_, PCC_700‐R_, and PCC_900‐R_.

**Figure 3:**
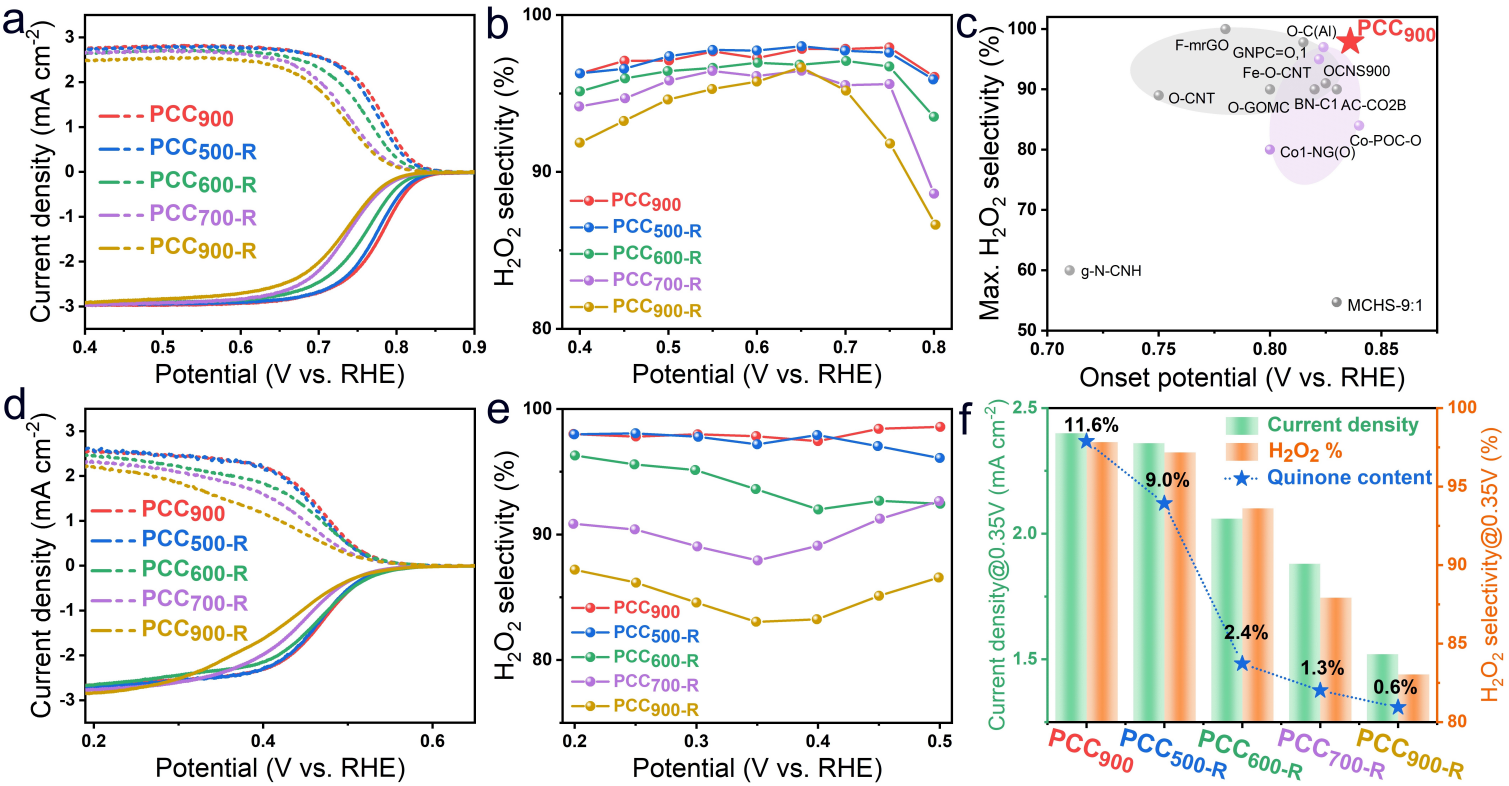
a) ORR polarization curves of disk current density (solid line) and ring current density (dash line) of PCC_900_, PCC_500‐R_, PCC_600‐R_, PCC_700‐R_, PCC_900‐R_ in 0.1 M KOH. b) Calculated H_2_O_2_ selectivity (H_2_O_2_ %). c) Comparison of the highest H_2_O_2_ selectivity and onset potentials for reported state‐of‐the‐art electrocatalysts. d) ORR polarization curves in 0.1 M PBS (pH 7). e) Calculated H_2_O_2_ selectivity (H_2_O_2_ %). f) The comparison of ring current density and H_2_O_2_ selectivity at 0.35 V vs. RHE, and quinone content obtained from CV results.

The wide pH adaptability would greatly promote the application of H_2_O_2_ electrocatalysts in different environments, such as H_2_O_2_ bleaching in alkaline conditions and bacteria killing in neutral conditions.^[3]^ The electrochemical performance of prepared materials is evaluated by using the three‐electrode rotating ring‐disk electrode (RRDE) system in O_2_‐saturated 0.1 M KOH and 0.1 M phosphate buffered solution (PBS, pH 7) electrolytes. Figure 3a displays that the PCC_900_ catalyst in 0.1 M KOH shows an onset potential of 0.83 V vs. RHE (0.1 mA cm^−2^) and the potential at 1 mA cm^−2^ is 0.79 V vs. RHE. Notably, H_2_O_2_ selectivity reaches 97.9 % at 0.75 V vs. RHE and it retains above 95 % in a large potential range of 0.4–0.8 V vs. RHE. The onset potential, H_2_O_2_ selectivity, and mass activity outperform most state‐of‐the‐art ORR catalysts including both carbon‐based and metal‐based catalysts for H_2_O_2_ electrocatalytic production (Figure 3c, S18 and Table S2). Interestingly, after re‐annealing treatment at 500 °C, the ORR performance of PCC_500‐R_ exhibits negligible changes compared with that of PCC_900_. When the re‐annealing temperature is further elevated from 600 °C to 900 °C, the onset potential and H_2_O_2_ selectivity both decline. Meanwhile, the kinetic current is determined by the Koutecky–Levich equation and Tafel plots (Figure S21a). The small Tafel slopes of PCC_900_ (38 mV dec^−1^) and PCC_500‐R_ (39 mV dec^−1^) reveal their rapid ORR kinetics. The increased Tafel slopes indicate that the ORR kinetic activity declines with increasing re‐annealing temperature, due to the gradual removal of the quinone groups, as evidenced by NEXAFS and CV measurements. Although most of the quinone groups are removed after re‐annealing at 900 °C, PCC_900‐R_ still displays good H_2_O_2_ selectivity (>90 %). This is likely caused by the residual quinone group and the intrinsic ORR activity of carbon catalysts in alkaline media.

Similar behavior is also observed in the neutral electrolyte. Compared with their performance in alkaline media, carbon catalysts usually exhibit sluggish kinetics in neutral media due to the lack of hydroxyl groups.^[24]^ Nevertheless, PCC_900_ presents an onset potential of 0.55 V vs. RHE and high H_2_O_2_ selectivity of over 95 % in the potential range of 0.2–0.5 V vs. RHE, surpassing most 2 e^−^ ORR electrocatalysts (Table S3). Notably, PCC_500‐R_ displays almost the same LSV curves and H_2_O_2_ selectivity compared with PCC_900_ (Figure 3d and e). Figure 3f displays the ring current density and H_2_O_2_ selectivity of PCC_900_, PCC_500‐R_, PCC_600‐R_, PCC_700‐R_, and PCC_900‐R_ at 0.35 V vs. RHE, as well as the quinone content calculated according to the area integral ratio of quinone redox peaks in CV curves in N_2_‐saturated 0.1 M PBS solution. After re‐annealing at 900 °C, the ring current density and H_2_O_2_ selectivity at 0.35 V vs. RHE decline to 1.53 mA cm^−2^ and 83.4 % for PCC_900‐R_, respectively. The current density and H_2_O_2_ selectivity are positively correlated with the quinone group content, indicating the quinone group is the active site for the 2 e^−^ ORR pathway. Furthermore, the Tafel plots reveal that the kinetic activity declines with decreasing quinone content (Figure S21b). Moreover, due to the exposure of the outer surface and the absence of porous structure, quinone groups are absent on annealed TA materials without the protection of SiO_2_ template, presenting very poor electrocatalytic performance (Figure S22).

Furthermore, Raman spectroscopy, SEM, and N_2_ adsorption‐desorption measurements are performed to rule out the influence of defects, morphology, and surface area during the re‐annealing process. As displayed in Figure S23a, close *I*_D_/*I*_G_ ratios reveal similar defect densities in PCC_500‐R_, PCC_600‐R_, PCC_700‐R_, and PCC_900‐R_, excluding changes in defect density as a potential cause of the observed change of property differences.^[25]^ The porous structure remains after re‐annealing treatment (Figure S23b–d), further indicating that the difference in electrochemical performance mainly results from the loss of quinone groups on the PCC surface. Moreover, considering alkaline treatment has been reported to introduce OFGs on the carbon surface,^[13]^ hydrofluoric acid (HF) treatment is also employed to remove the silica nanospheres for comparison (Figure S24). The CV, LSV curves, and H_2_O_2_ % selectivity are nearly identical, thus eliminating the effect of alkaline treatment on the formation of quinone groups as another potential explanation for the improved performances. In addition, similar performance is observed when employing different carbon precursors, including gallic acid and L‐ascorbic acid (Figure S25). This further reveals that silica nanospheres play crucial roles in the formation of quinone groups which are independent of carbon precursors.

Considering the potential for practical applications, the stability of the ORR performance on PCC_900_ is evaluated by an accelerated durability test (ADT). As displayed in Figure S26a–d, after 10 000 cycles, the activity and H_2_O_2_ selectivity are both largely maintained in neutral and alkaline electrolytes, indicating its high stability. Moreover, the catalyst possessed excellent aging‐resistant properties. The ORR performance of PCC_900_ does not show any change even after storage in air for 6 months (Figure S26e–f), which is significantly improved compared with metal‐doped catalysts.^[6b]^

Next, we examine the practical H_2_O_2_ electrocatalytic production in a three‐electrode H‐type cell with the catalyst loading of 0.1 mg cm^−2^ on a hydrophobic carbon paper. The H_2_O_2_ yield is calculated through a ceric sulfate (Ce(SO_4_)_2_) titration method (Figure S27).^[5]^ The working electrode potential is set at 0.564 V vs. RHE in 0.1 M KOH and 0.21 V vs. RHE in 0.1 M PBS, respectively. As displayed in Figure S28a and d, a steady current density is observed and the accumulated H_2_O_2_ yield increases with the reaction time. Within 1 h, the accumulated H_2_O_2_ yields reach 170 μmol in 0.1 M KOH (at 0.56 V vs. RHE) and 136 μmol H_2_O_2_ in 0.1 M PBS (at 0.21 V vs. RHE), of which the production rates are 28.28 mmol g_catalyst_^−1^ cm^−1^ min^−1^ and 22.68 mmol g_catalyst_^−1^ cm^−1^ min^−1^, respectively (Figure S28c and f). In addition, the Faradaic efficiency is over 90 % in the whole potential range. This performance outperforms most of the reported metal‐based catalysts and demonstrates great potential for practical applications (Table S4).

**Figure 4:**
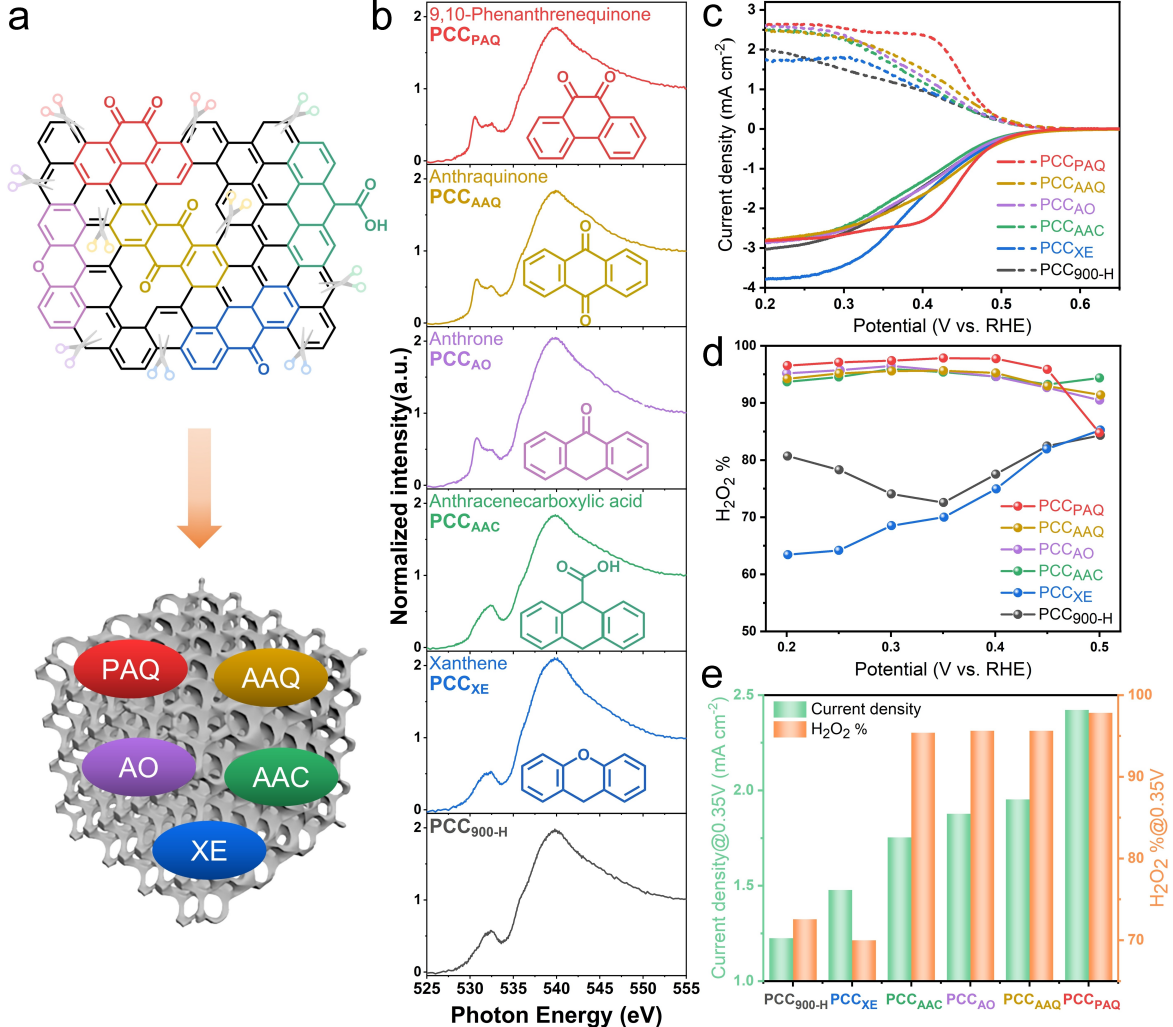
a) Structures of typical OFGs and aromatic organic molecules with isolated OFGs including C=O, COOH, and C−O−C. b) O K‐edge NEXAFS spectra of PCC_900‐H_, PCC_XE_, PCC_AAC_, PCC_AO_, PCC_AAQ_, and PCC_PAQ_ measured on the gold substrate. c) ORR polarization curves of disk current density (solid line) and ring current density (dash line) of PCC_900‐H_, PCC_XE_, PCC_AAC_, PCC_AO_, PCC_AAQ_, and PCC_PAQ_ in 0.1 M PBS (pH 7). d) Calculated H_2_O_2_ selectivity (H_2_O_2_ %). e) Ring current density and H_2_O_2_ selectivity at 0.35 V vs. RHE.

On the other hand, even though OFGs have been widely reported to be highly effective for H_2_O_2_ electrosynthesis, the identification of specific active OFGs remains a big challenge.[^9a^, ^11^] Generally, multiple OFGs tend to exist on carbon materials simultaneously and it is difficult to accurately discriminate them by characterization techniques such as XPS and FTIR. Consequently, to further investigate the specific role of different OFGs, a molecule‐mimic strategy is employed. Different aromatic molecules (AMs) with specific oxygen species including anthracenecarboxylic acid (AAC), xanthene (XE), anthrone (AO), anthraquinone (AAQ), and 9,10‐phenanthrenequinone (PAQ) are decorated on the PCC_900‐H_ support via strong noncovalent π‐π interactions by a simple solvothermal method,^[26]^ as shown in Figure 4a.

COOH and C−O−C oxidized carbon catalysts are simulated by AAC and XE decorated PCC_900‐H_, and denoted as PCC_AAC_ and PCC_XE_, respectively. Similarly, PCC_AO_, PCC_AAQ_, and PCC_PAQ_ are applied to simulate the C=O oxidized carbon catalysts and further elucidate the effect of C=O positions for the 2 e^−^ ORR process. Prior to the solvothermal process, PCC_900‐H_ is prepared by re‐annealing the PCC_900_ powder in a 5 % H_2_/N_2_ mixture gas at 900 °C for 4 h to remove the surface oxygen species. As displayed in Figure 4b and Figure S29, the peak at ≈530.7 eV corresponding to the π* (C=O) is absent in PCC_900‐H_, PCC_AAC,_ and PCC_XE_. The intensity of the π* (C=O) peak is clear for PCC_AO_ with single C=O, and PCC_AAQ_ and PCC_PAQ_ with two C=O configurations in the AMs, indicating the successful decoration of AMs on the PCC_900‐H_ substrate.

The electrochemical performance of AM‐decorated samples is then evaluated using the RRDE in neutral and alkaline electrolytes as displayed in Figure 4c and Figure S30–S31. Quinone molecules present unique and obvious redox peaks in N_2_‐saturated CV curves, which are not observed for PCC_AAC_ and PCC_XE_ (Figure S31a). It has been reported that the redox peak position of quinone molecules is dependent on their oxygen configurations.[^26a^, ^27^] In this work, the redox peak of PCC_PAQ_ appears at around 0.38–0.45 V vs. RHE in 0.1 M PBS, close to the redox peak position of PCC_900_ and PCC_500‐R_. In contrast, the redox peak of PCC_AO_ and PCC_AAQ_ is located at around 0.03–0.12 V vs. RHE. Similarly, in 0.1 M KOH, PCC_PAQ_ presents more positive redox peaks at around 0.39–0.45 V vs. RHE, close to that of PCC_900_ and PCC_500‐R_ (0.40–0.50 V vs. RHE), whereas the peaks are located at 0.05–0.12 V vs. RHE for PCC_AO_ and PCC_AAQ_. Considering the coincidence between the redox peak position of PCC_AAQ_ and PCC_900_, it is believed that the oxygen species on the PCC_900_ likely consists of a 9,10‐phenanthrenequinone configuration. As for the ORR performance in 0.1 M PBS, in contrast to PCC_900‐H_, AM‐decorated materials with C=O groups, including PCC_AO_, PCC_AAQ_, and PCC_PAQ_, exhibit highly improved H_2_O_2_ selectivity and activity (Figure 4c–e). The ring current density at 0.35 V vs. RHE is 1.23 mA cm^−2^, 1.88 mA cm^−2^, 1.95 mA cm^−2^, 2.42 mA cm^−2^, and the H_2_O_2_ selectivity is 72.57 %, 95.63 %, 95.64 %, 97.83 % for PCC_900‐H_, PCC_AO_, PCC_AAQ_, and PCC_PAQ_, respectively, indicating that quinone species or heterocyclic ketone species render high electrocatalytic properties to carbon materials. Remarkably, the PCC_PAQ_ exhibits the best H_2_O_2_ selectivity and activity among these AM‐decorated materials. The H_2_O_2_ selectivity is above 95 % in a wide potential range of 0.2–0.45 V vs. RHE and the Tafel slope is 52 mV dec^−1^ (Figure S31b), revealing outstanding properties for H_2_O_2_ generation of the 9,10‐phenanthrenequinone configuration. Moreover, an improved H_2_O_2_ selectivity on PCC_AAC_ demonstrates that the COOH configuration also contributes to the 2 e^−^ ORR pathway.^[28]^ However, PCC_XE_ with the C−O−C configuration tends to follow a 4 e^−^ ORR pathway, which is in contradiction to previously reported works.^[9a]^ The ORR performance in 0.1 M KOH is displayed in Figure S30. PCC_PAQ_ still exhibits the best H_2_O_2_ selectivity and the highest ring current density. Unfortunately, due to the intrinsic activity and selectivity of porous carbon, as well as the rapid ORR kinetics in the alkaline electrolyte,^[29]^ it is difficult to observe obvious performance differences compared with that in the neutral electrolyte. To further clarify the superior H_2_O_2_ electrocatalytic production performance of the PAQ molecule, AMs are also decorated on the H_2_‐annealed carbon black (CB) and their ORR performance is displayed in Figure S32. CB_PAQ_ achieves the highest H_2_O_2_ selectivity and activity in both alkaline and neutral electrolytes. Besides, the ORR performance of different AM‐decorated CB_900‐H_ materials is akin to that of the AM‐decorated PCC_900‐H_ materials.

**Figure 5:**
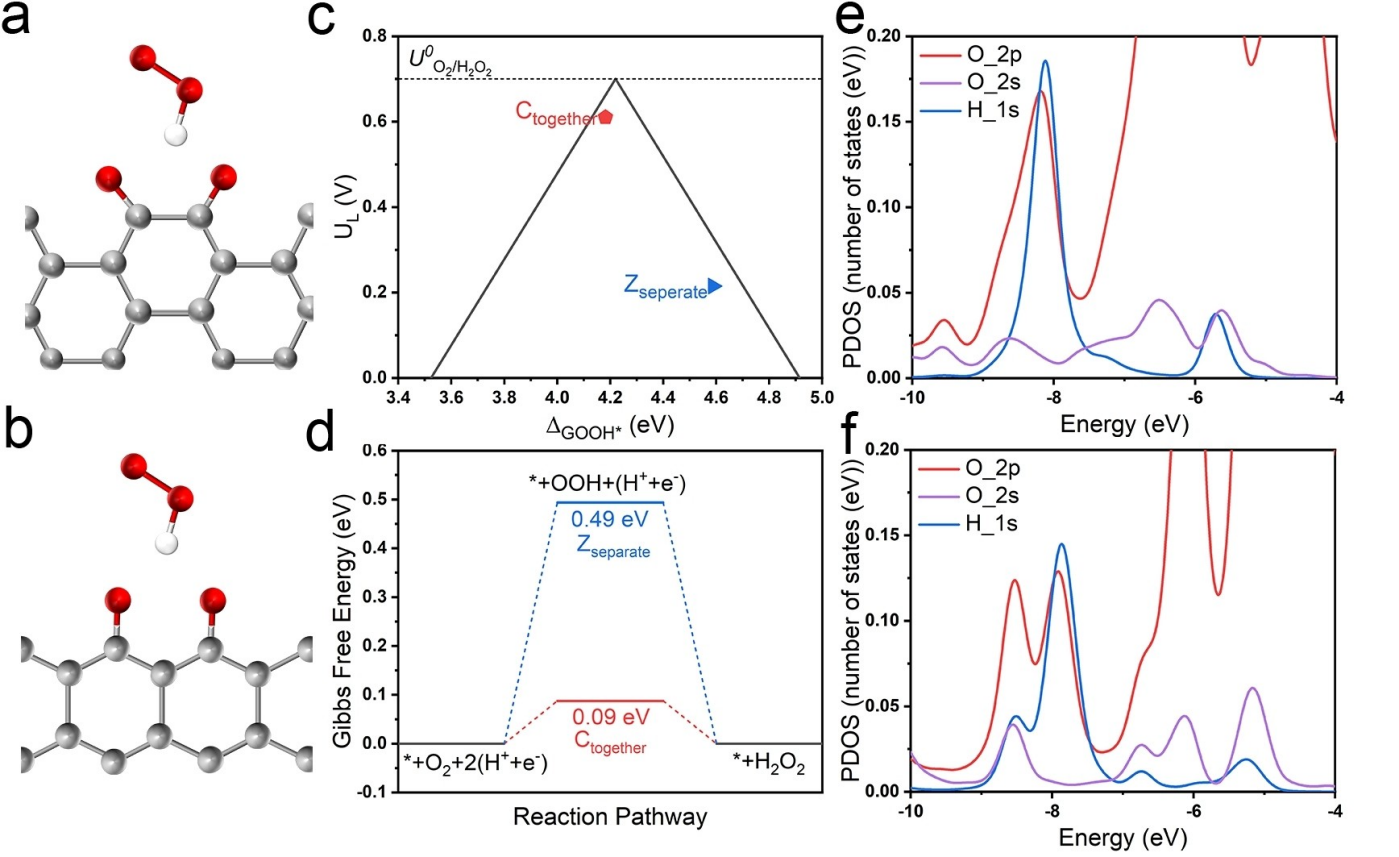
The optimized structures of a) C_together_ and b) Z_separated_ forms. The interaction of the OOH* species is also displayed. The grey, red, and white balls are C, O, and H‐atoms, respectively. For clarity, only the active site has been shown. c) Calculated activity‐volcano plot for 2 e^−^ ORR. The limiting potential is plotted as a function of Δ*G*_OOH*_. The dashed black line represents the equilibrium potential for the theoretical 2 e^−^ ORR. d) Gibbs free energy diagram of 2 e^−^ ORR on C_together_ and Z_separated_ at *U*=0.7 V. The partial density of states for e) C_together_ and f) Z_separated_ systems.

Density functional theory calculations are applied to further investigate the formation of quinone groups and scrutinize their active sites. In contrast to other OFGs, carbonyl or ketone groups have been widely proven to be prone to H_2_O_2_ electrocatalytic production.[^11^, ^12^] Moreover, both ketone and quinone groups exhibit high decomposition temperatures.^[14]^ Herein, two different graphene nanoribbon (GNR) models are employed to understand the difference between the ketone and quinone groups: (i) two adjacent ketone groups (quinone) are decorated on the same phenyl ring of a chair GNR, denoted as C_together_ (Figure 5a), and (ii) two separate ketone groups are decorated on two adjacent phenyl rings of a zigzag GNR, referred to as Z_separated_ (Figure 5b). In all these models, ketone groups are decorated on the edge of GNR.^[30]^

The formation energy per atom (*E*_formation/atom_) of these two models is first calculated, which reveals that the C_together_ model (24.057 kJ mol^−1^) is more stable than the Z_separated_ model (22.749 kJ mol^−1^). This could partially elucidate the reason that more stable quinone groups remain after the high‐temperature annealing process, instead of ketone or other OFGs.

Furthermore, the adsorption energy of the OOH* (Δ*G*_OOH*_) is used as a key descriptor to evaluate the electrocatalytic activity of C_together_ and Z_separated_ models.^[9]^ The calculated limiting potential (*U_L_*), which is generally defined as the maximum potential at which the reduction of O_2_ to OOH* and subsequent reduction of OOH* to H_2_O_2_ is downhill in free energy, is displayed in Figure 5c as a function of Δ*G*_OOH*_. On the left side of the volcano plot, the stronger adsorption of OOH* on the active site exhibits its higher activity, whereas the active site with weaker adsorption of OOH* is located on the right side which realizes higher selectivity but lower activity. An ideal catalyst is supposed to locate or approach the peak of the volcano plot with an ideal Δ*G*_OOH*_ of 4.22 eV. The C_together_ and Z_separated_ models reveal a Δ*G*_OOH*_ of 4.18 eV and 4.59 eV, respectively, indicating the superiority of chair‐form quinone groups for the 2 e^−^ ORR process. Furthermore, more intuitive results could be obtained from the free energy diagram (Figure 5d) in which C_together_ model presents a much lower energy barrier in the 2 e^−^ ORR process in contrast to that of Z_separated_. This is in good agreement with the experimental results that PCC_PAQ_ exhibits the best onset potential and H_2_O_2_ selectivity among all AM‐decorated catalysts.

To further understand the superior adsorption of Δ*G*_OOH*_ on the C_together_ model, we consider the partial density of states (PDOS) of the Z_separated_ and the C_together_ systems (Figure S33). As expected, the PDOS is mainly contributed by the O *2p* states. Traces of H 1*s* states from the OOH* species can be seen around the Fermi energy (*E*_F_). However, as we move to lower energy levels, a significant contribution from the H 1s states, overlapping with the O 2*s* and 2*p* states can be seen (Figure 5e and f). In the case of the Z_separated_, the overlapping region is between −8.612 eV to −5.563 eV, while it is between −8.646 eV to −5.672 eV in the C_together_ system. The overlapping region of the H 1*s* states with the O 2*s* and 2*p* states is larger than that in the C_together_, indicating a stronger H‐bonding. To further quantify this, we calculate the area of the overlapping region using $O_{A}=\int_{-a}^{-b}sdE$ (where $O_{A}$ is the overlapping area, *‐a* and *‐b* are the lower and upper limit of the overlapping region and *s* is the contribution of the H *s*‐and O *p*‐states), which reveals 0.115 and 0.100 number of states for C_together_ and Z_separated_ systems, respectively, for the H *s*‐orbitals, and 0.544 and 0.372 number of states for the C_together_ and Z_separated_ systems, respectively, for the O *p‐*states.^[31]^ This clearly shows stronger H‐bonding between OOH and C_together_ as compared to the Z_separated_.

## Conclusion

In conclusion, we propose a facile template‐protected strategy to synthesize highly efficient quinone‐doped porous carbon catalysts for H_2_O_2_ electrocatalytic production. The positive onset potential and high selectivity in both alkaline and neutral electrolytes outperform most state‐of‐the‐art 2 e^−^ ORR catalysts, exhibiting promising practical potentials. The outstanding performance is attributed to the high content of quinone groups, and the positive correlation between the content of quinone groups and the performance of electrosynthesis of H_2_O_2_ is confirmed by NEXAFS, CV, and LSV measurements. For O K‐edge NEXAFS analysis, the importance of sample substrate is highlighted to get rid of the interference, as well as the standard measurement and analysis procedures, which provides significant guidance for future users. The role of quinone groups is further revealed by investigating the 2 e^−^ ORR performance of distinctive oxygen‐containing aromatic molecules on different carbon supports using a molecule‐mimic strategy. This contributes to the understanding of the role of different OFGs and provides new insights into the design and scrutiny of active sites for a variety of electrocatalysts.

## Conflict of interest

The authors declare no conflict of interest.

1

## Supporting information

As a service to our authors and readers, this journal provides supporting information supplied by the authors. Such materials are peer reviewed and may be re‐organized for online delivery, but are not copy‐edited or typeset. Technical support issues arising from supporting information (other than missing files) should be addressed to the authors.

Supporting Information

## Data Availability

The data that support the findings of this study are available from the corresponding author upon reasonable request.
