# Supplementary material for: Spectroscopic Identification of Active Sites of Oxygen‐Doped Carbon for Selective Oxygen Reduction to Hydrogen Peroxide
Source: Angew Chem Int Ed Engl. 2023 Apr 18;62(21):e202303525. doi: 10.1002/anie.202303525 (PMC10947142; doi:10.1002/anie.202303525)
Supplement: Supplementary file 1 — Supporting Information [file ANIE-62-0-s001.pdf]

Angewandte Chemie
Eine Zeitschrift der Gesellschaft Deutscher Chemiker

Supporting Information

Spectroscopic Identification of Active Sites of Oxygen-Doped Carbon for Selective Oxygen Reduction to Hydrogen Peroxide

L. Liu, L. Kang, A. Chutia, J. Feng, M. Michalska, P. Ferrer, D. C. Grinter, G. Held, Y. Tan, F. Zhao, F. Guo, D. G. Hopkinson, C. S. Allen, Y. Hou, J. Gu, I. Papakonstantinou, P. R. Shearing, D. J. L. Brett, I. P. Parkin, G. He**

Supporting Information
©Wiley-VCH 2021
69451 Weinheim, Germany

Spectroscopic Identification of Active Sites of Oxygen-Doped Carbon for Selective Oxygen Reduction to Hydrogen Peroxide

Longxiang Liu[a], Liqun Kang[b], Arunabhiram Chutia[c], Jianrui Feng[a], Martyna Michalska[d], Pilar Ferrer[e], David C. Grinter[e], Georg Held[e], Yeshu Tan[a], Fangjia Zhao[a], Fei Guo[a], David G. Hopkinson[f], Christopher S. Allen[f], [g], Yanbei Hou[h], Junwen Gu[a], Ioannis Papakonstantinou[d], Paul R. Shearing[i], Dan J.L. Brett[i], Ivan P. Parkin*[a], Guanjie He*[a],[i]

Abstract: The electrochemical synthesis of hydrogen peroxide (H_2O_2) *via* a two-electron ($2e^-$) oxygen reduction reaction (ORR) process provides a promising alternative to replace the energy-intensive anthraquinone process. Herein, we develop a facile template-protected strategy to synthesize a highly active quinone-rich porous carbon catalyst for H_2O_2 electrochemical production. The optimized PCC_{900} material exhibits remarkable activity and selectivity, of which the onset potential reaches 0.83 V *vs.* reversible hydrogen electrode in 0.1 M KOH and the H_2O_2 selectivity is over 95 % in a wide potential range. Comprehensive synchrotron-based near-edge X-ray absorption fine structure (NEXAFS) spectroscopy combined with electrocatalytic characterizations reveals the positive correlation between quinone content and $2e^-$ ORR performance. The effectiveness of chair-form quinone groups as the most efficient active sites is highlighted by the molecule-mimic strategy and theoretical analysis.

Table of Contents

Experimental Procedures 4
Figure S1 7
Figure S2 8
Figure S3 9
Figure S4 10
Figure S5 11
Figure S6 12
Figure S7 13
Figure S8 14
Figure S9 15
Figure S10 16
Figure S11 17
Supporting Note 1 17
Figure S12 18
Figure S13 19
Supporting Note 2 19
Supporting Note 3 20
Supporting Note 4 20
Figure S14 21
Supporting Note 5 21
Figure S15 22
Supporting Note 6 22
Figure S16 23
Figure S17 24
Figure S18 25
Figure S19 26
Figure S20 27
Figure S21 28
Figure S22 29
Figure S23 30
Figure S24 31
Figure S25 32
Figure S26 33
Figure S27 34
Figure S28 35
Figure S29 36
Figure S30 37
Figure S31 38
Figure S32 39

Figure S33 ... 40

Table S1 ... 41

Table S2 ... 42

Table S3 ... 43

Table S4 ... 44

Table S5 ... 45

References ... 46

Author contributions ... 47

Experimental Procedures

Chemicals and materials

Tannic acid (TA), silica nanosphere (fumed powder, 5-20nm), sodium hydroxide (NaOH), potassium hydroxide (KOH), potassium phosphate monobasic (KH_2PO_4), potassium phosphate dibasic (K_2HPO_4), nafion perfluorinated resin solution (5 wt%), hydrochloric acid (HF), L-ascorbic acid (LAC), gallic acid (GA), xanthene (XE), anthrone (AO), 9-anthracenecarboxylic acid (AAC), anthraquinone (AAQ), 9,10-phenanthrenequinone (PAQ), and absolute ethanol were purchased from Sigma-Aldrich (UK) Co., Ltd.

Materials synthesis

Synthesis of PCC. To prepare the porous carbon catalyst (PCC), 0.5 g tannic acid and 0.9 g silica nanosphere were dissolved in 10 mL deionized water and ultrasonicated for 20 min. Subsequently, the mixed solution was frozen immediately using liquid nitrogen and freeze-dried for 48 h. The dried powder was annealed at 900 °C for 2 h under an Ar atmosphere with a heating rate of 5 °C/min in a tube furnace. After that, the carbonized product was dispersed in 100 mL of 2 M NaOH solution and heated at 90 °C for 12 h in the Teflon-lined stainless-steel autoclave. To remove the silica template completely, the solution was decanted and fresh NaOH solution was added and heated at 90 °C for another 12 h. Finally, the product was washed with excessive deionized water using vacuum filtration. The as-prepared sample was named as PCC_{900}. Meanwhile, 10 wt% hydrofluoric acid (HF) was also used to remove the silica template by stirring the carbonized product (2 g 100 mL^{-1}) for 5 h and washing with excessive deionized water. The HF-treated sample was denoted as $PCC_{900\text{-}HF}$. The samples with different ratios of tannic acid to silica nanosphere, namely 0.5 g:0 g, 0.5 g:0.5 g, 0.5 g:0.7 g, and 0.5 g:1.1 g, were prepared by the same process and denoted as $PCC_{5\text{-}0}$, $PCC_{5\text{-}5}$, $PCC_{5\text{-}7}$, and $PCC_{5\text{-}11}$, respectively.

Synthesis of $PCC_{N\text{-}R}$ (N=500, 600, 700, 900). PCC_{900} was then re-annealed at 500°C, 600°C, 700°C, 900°C for 1 h with a heating rate of 10 °C/min in an Ar atmosphere. Re-annealed samples were denoted as $PCC_{500\text{-}R}$, $PCC_{600\text{-}R}$, $PCC_{700\text{-}R}$, $PCC_{800\text{-}R}$, and $PCC_{900\text{-}R}$, respectively.

Synthesis of PCC_{AMs} (AMs=PAQ. XE, AAC, AO, AAQ). $PCC_{900\text{-}H}$ was prepared through re-annealing PCC_{900} at 900°C for 4 h with a heating rate of 10 °C/min in 5 % H_2/N_2 mixture gas atmosphere. PCC_{PAQ} was prepared through a simple solvothermal process. 30 mg $PCC_{900\text{-}H}$ and 20 mg 9,10-phenanthrenequinone (PAQ) were first ultrasonicated in 30 mL ethanol for 20 min. The solution was heated at 100 °C for 12 h in the Teflon-lined stainless-steel autoclave. Afterwards, the precipitates were washed with excessive ethanol and deionized water by centrifugation method and freeze-dried for 48 h. PCC_{XE}, PCC_{AAC}, PCC_{AO}, and PCC_{AAQ} were prepared using different aromatic organic molecules, namely xanthene (XE), 9-anthracenecarboxylic acid (AAC), anthrone (AO), and anthraquinone (AAQ), respectively. The same ratios of AM to $PCC_{900\text{-}H}$, as well as the same preparation process, including the step of excessive rinsing to remove unabsorbed AM, are used to exclude the effect of loading amount.

Materials characterization

The structure and morphology of the samples were observed on scanning electron microscope (SEM, Carl Zeiss EVO MA10), transmission electron microscopy (JEOL, JEM-2100), and scanning transmission electron microscopy (JEOL ARM300CF, beamline E02 of Diamond Light Source (UK)). X-ray photoelectron spectroscopy (XPS, Thermo scientific K-alpha photoelectron spectrometer) was conducted to determine the elements and surface functional groups. Shirley Background and GL(30) line shape were used for deconvolution analysis using CasaXPS software. The atomic concentrations were determined by the ratio of peak area and relative sensitivity factor (peak area / R.S.F) and the RSF was from the default CasaXPS library. Nitrogen adsorption–desorption isotherms were recorded at 77 K using a QUADRASORB evo Instruments (Quantachrome, USA). Fourier-transform infrared spectroscopy (FTIR) was performed in transmission mode (with KBR, Shimadzu IRTracer-100) and Attenuated Total Reflection (ATR) mode (Geranium crystal, Bruker ALPHA II FTIR). The specific surface area of samples was determined according to the Brunauer–Emmett–Teller (BET) method. Raman spectroscopy (514.5 nm laser, Renishaw) was carried out by placing samples on a glass slide. Ultraviolet–visible (UV) spectroscopy was recorded on a Shimadzu UV-2700 spectrophotometer. The near-edge X-ray absorption fine structure (NEXAFS) experiments were performed at Branch B of the VerSoX beamline B07 of Diamond Light Source (UK)[18]. The schematic illustration of the beamline end-station set-up of B07-B@Diamond Light Source is displayed in Figure S9. Data were collected at C K-edge and O K-edge in total electron yield (TEY) mode under vacuum ($<1\times10^{-7}$ mbar). The samples were prepared by either pressing into indium plates or by drop-casting onto gold-coated silicon wafers using deionized water as a solvent. 5 repetitions of NEXAFS scans were collected and averaged to improve the signal-to-noise ratio for each sample. The energy shift was calibrated based on the feature of incident beam current (I_0). Arctangent step function and Gaussian peak functions were used to perform peak fitting on Athena software[1]. More details of the data process are listed in supporting notes 1-6.

Electrochemical tests

All the electrochemical tests were carried out in a three-electrode cell system controlled by a Gamry potentiostat. The RRDE working electrode (Pine E7R9) comprises a glassy carbon rotation disk electrode (0.2475 cm^2 area) and a Pt ring (0.1866 cm^2 area) with a theoretical collection efficiency (N) of 37%. An Ag/AgCl electrode and a graphite rod were used as the reference electrode and the counter electrode, respectively. 0.3 μm, 0.1 μm, and 0.05 μm Al_2O_3 polishing suspension were successively used to clean the working electrode. To prepare the ink, 2.5 mg of catalyst was dispersed in 1 mL of ethanol and 30 μL of 5.0 wt% Nafion solution. After

ultrasonication for 30 min, 10.0 μL suspension was drop-casted onto the disk electrode on the spinning stage and dried at room temperature.

The oxygen reduction activity and selectivity of the samples were evaluated by RRDE measurements in oxygen-saturated 0.1 M KOH and 0.1 M phosphate buffered solution (PBS, pH=7). Cyclic voltammetry (CV) was first performed at a scan rate of 50 mV s^{-1} between 0.164 and 0.964 V in O_2-saturated electrolytes until a steady CV was obtained. After that, RRDE measurement was carried out in O_2-saturated electrolyte between 0.164 and 0.964 V at a scan rate of 5 mV s^{-1} under 1600 rpm. The potential of the Pt ring electrode was set to 1.2 V. To eliminate the formation of PtO_x, chronoamperometry was carried out at 0 V for 60 s before the test. The electrochemical impedance spectroscopy (EIS) was measured for *iR* correction.

All electrode potentials were calibrated to RHE using the Eq. (1):

$$E_{RHE} = E_{Ag/AgCl} + 0.197 + 0.059\ pH - 0.95\ iR \quad (1)$$

The H_2O_2 selectivity was determined based on Eq. (2).

$$H_2O_2\ \% = 200 \times \frac{I_{Ring}/N}{I_{Disk} + I_{Ring}/N} \quad (2)$$

where I_{Ring} is the ring current, I_{Disk} is the disk current and *N* is the collection efficiency. The experimental *N* was determined by the redox reaction of $[Fe(CN)_6]^{4-}/[Fe(CN)_6]^{3-}$, where the reactions taking place on the disk and ring are[2]:

$$\text{Disk: } Fe(CN)_6^{\ 3-} + e^- \rightarrow Fe(CN)_6^{\ 4-} \quad (3)$$

$$\text{Ring: } Fe(CN)_6^{\ 4-} \rightarrow Fe(CN)_6^{\ 3-} + e^- \quad (4)$$

The electrode was rotated at 1600 rpm in N_2-saturated 0.1 M KOH and 0.004 M $K_3Fe(CN)_6$ solution. Bipotential chronoamperometry method was performed and the voltage of the ring and disk was set to 1.5 V and 0.1 V, respectively. The measurement lasted 60 s and the disk current (I_d) and ring current (I_r) of the last 10 s were averaged. The background current of the ring (I_{rbg}) was determined by repeating the same measurement except for disconnecting the disk. The *N* value was calculated according to the following Eq. (5).

$$N = \frac{I_r - I_{rbg}}{I_d} \quad (5)$$

The result of the calibration of N was shown in Figure S19. And the experimental N was determined to be 0.375.

Tafel slopes were evaluated according to the Koutecky-Levich equation (Eq. 6):

$$\frac{1}{J_{H_2O_2}} = \frac{1}{J_{K,\ H_2O_2}} + \frac{1}{J_{L,\ H_2O_2}} \quad (6)$$

where $J_{H_2O_2}$ is the measured ring current density. $J_{L,\ H_2O_2}$ is the theoretical limiting current of the $2e^-$ ORR process, which is determined to be 2.9 mA cm^{-2}. $J_{K,\ H_2O_2}$ is the kinetic current of H_2O_2[3].

Bulk electrolysis

Bulk electrochemical H_2O_2 production was carried out in a three-electrode H-cell separated by Nafion 117 membrane (Sigma-Aldrich). Prior to use, Nafion 117 membrane was immersed in 3 wt % H_2O_2, ultrapure water, 1 M H_2SO_4, and ultrapure water at 80°C for 1 h successively and washed with excessive ultrapure water. Each chamber of H-cell contained 20 mL 0.1 M KOH or 0.1 M PBS solution. 0.1 mg PCM_{900} catalyst was drop-casted on the PTFE-coated carbon paper (1 cm × 1 cm) and used as the working electrode. An Ag/AgCl electrode and a graphite rod were used as the reference electrode and the counter electrode, respectively. Chronoamperometry measurements (without *iR*-correction) were performed at a potential of ~~0.3~~ vs Ag/AgCl (~~0.11~~ V vs RHE in 0.1 M PBS, and 0.564 V vs RHE in 0.1 M KOH) for 1 h on a magnetic stirrer at a 300 rpm stirring rate. During the measurement, steady oxygen gas flow was purged into the working electrode compartment electrolyte to ensure the saturated concentration of dissolved O_2 gas.

Cerium sulfate $Ce(SO_4)_2$ titration method was used to measure the produced H_2O_2 concentration. The yellow solution of Ce^{4+} can be reduced to colorless Ce^{3+} by H_2O_2 based on Eq. (7). The concentration of H_2O_2 can be calculated by Eq. (8). The Faradaic efficiency for H_2O_2 production was calculated by Eq. (9). Different concentration of $Ce(SO_4)_2$ (up to 0.5 mM) were prepared in 0.5 M H_2SO_4 solution. The absorbance intensity at 319 nm in UV spectroscopy of known concentration (0, 0.05, 0.1, 0.15, 0.2, 0.3, 0.4, 0.5 mM) of Ce^{4+} solution was linearly calibrated, as shown in Figure S 24.

For bulk electrolysis in 0.1 M KOH, 50 μL of solution in the working electrode chamber was sampled and injected immediately into 4.95 mL of 0.482 mM Ce^{4+} solution for every 10 min. For bulk electrolysis in 0.1 M KOH, 100 μL of solution in the working electrode chamber was sampled and injected immediately into 4.90 mL of 0.463 mM Ce^{4+} solution every 10 min.

$$2Ce^{4+} + H_2O_2 \rightarrow 2Ce^{3+} + 2H^+ + O_2 \quad (7)$$

$$M\,(H_2O_2) = 1/2\ M\,(Ce^{4+}) \quad (8)$$

where $M\,(Ce^{4+})$ is the mole of consumed Ce^{4+}.

$$\text{Faradaic efficiency (\%)} = \frac{2 \times 96485 \times M(H_2O_2)}{\int_0^t I\,dt} \times 100\% \quad (9)$$

where $\int_0^t I\,dt$ is the cumulative charge.

Computational methods

We used the Vienna Ab Initio Simulation Package (VASP) to perform periodic spin-polarized periodic density functional theory calculations[4]. The projector augmented wave (PAW) method was employed and for the expansion of the plane-wave basis sets, the cut-off energy was set to 450 eV, which gave bulk energies converged to within 10^{-5} eV[5]. We chose a convergence criterion of 0.01 eV $Å^{-1}$ for the structural optimizations and a gamma centered k-point grid of 5×3×1 was employed. The Perdew-Burke-Ernzerhof (PBE) version of the exchange and correlation function was used to relax the structures and to perform the total energy calculations[6]. In this study, we also included Grimme's dispersion correction (DFT+D3) as dispersive effects may play a significant role in these systems under investigation[7]. The zigzag graphene nanoribbon (GNRs) models were modeled using the bulk graphite structure with a calculated energy minimized lattice constant of a=b= 2.468 Å and c = 6.823 Å (Exp. A=b=2.461 Å and c = 6.709 Å)[8]. For all the calculations, in the direction perpendicular to the surface, we used a vacuum gap of ~15 Å, which is sufficient to eliminate any spurious interactions along the z-axis.

The adsorption of the OOH* species on the GNRs models may lead to spurious dipole moment, which was taken into account by using the methods implemented in VASP according to the procedures of Neugebauer et al[9]. The charges on various atoms were obtained using the Bader charge analysis as implemented by Henkelman and coworkers [10].

The formation energy per atom ($E_{formation/atom}$) of the pristine GNRs with the quinone groups was calculated using:

$$\mathrm{E_{formation/atom}} = \left[\frac{\left\{l*\left(\frac{\mathrm{E_{graphite\ unit\ cell}}}{4}\right)+m*\left(\frac{\mathrm{E_{O_2}}}{2}\right)+n*\left(\frac{\mathrm{E_{H_2}}}{2}\right)\right\}-\mathrm{E_{pristine\ system}}}{(l+m+n)}\right] \quad (10)$$

where, $E_{graphite\ unit\ cell}$, E_{O_2}, E_{H_2} and $E_{pristine\ system}$ are the total energies of the graphite unit cell, oxygen molecule, hydrogen molecule, and energy of the pristine GNR models respectively. The *l, m,* and *n* are the total number of carbon, oxygen, and hydrogen atoms in the GNR models respectively.

The adsorption energy (E_{ad}) of the OOH* species on the GNR models was calculated using:

$$\mathrm{E_{ad}} = \mathrm{E_{GNR+OOH*}} - \mathrm{E_{pristine\ GNR}} - \mathrm{E_{OOH*}} \quad (11)$$

where, $E_{GNR+OOH*}$, $E_{pristine\ GNR}$ and E_{OOH*} are the total energies of the OOH* species adsorbed on GNR, pristine GNR model, and the OOH* models respectively.

For $2e^-$ ORR, there are generally two coupled electron and proton transfers:

$$O_2 + * + (H^+ + e^-) \rightarrow HOO* \quad (12)$$

$$HOO* + (H^+ + e^-) \rightarrow H_2O_2 + * \quad (13)$$

The computational hydrogen electrode (CHE) model was employed to evaluate the Gibbs reaction-free energy change (ΔG) for each step in the $2e^-$ ORR[11]. The free energy for each reaction intermediate is defined by Eq. (14):

$$\Delta G = \Delta E_{DFT} + \Delta E_{ZPE} - T\Delta S + eU + E_{sol} \quad (14)$$

ΔE_{DFT} is the adsorption energy calculated by DFT. ΔE_{ZPE} is the zero-point energy change and ΔS is the entropy change at 300 K. T, e, and U are the temperature, the number of electrons transferred and the electrode potential, respectively. The solvent correction is 0.45 eV obtained from previous literature[12]. The contributions of each component are listed in Table S5, where the entropies are obtained from NIST (National Institute of Standards and Technology). Considering the poor description of O_2 molecule in DF calculations, the free energies of H_2O_2 and H_2 were used as references to obtain the free energy of the O_2 molecule.

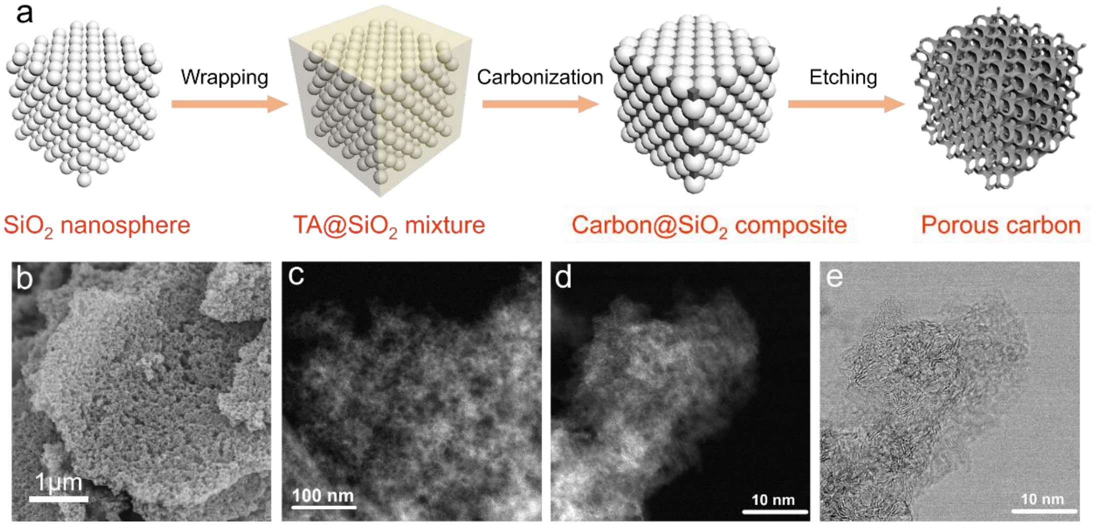

Figure S1. (a) Schematic representation of PCCs synthesis. (b) SEM, (c-d) HAADF-STEM, and (e) BF-STEM images of PCC_{900}.

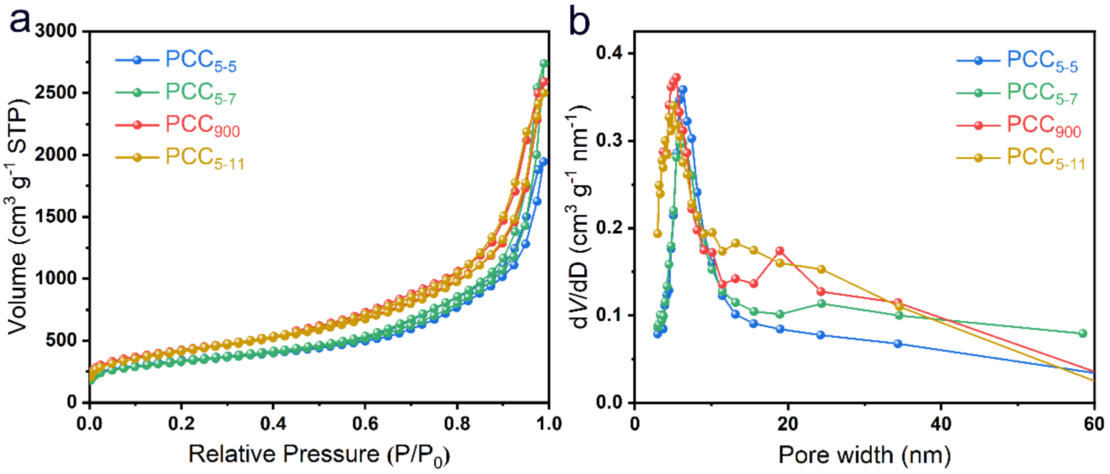

Figure S2. N_2 adsorption/desorption isotherms and (b) BJH pore size distribution of PCC_{5-5}, PCC_{5-7}, PCC_{900}, and PCC_{5-11}.

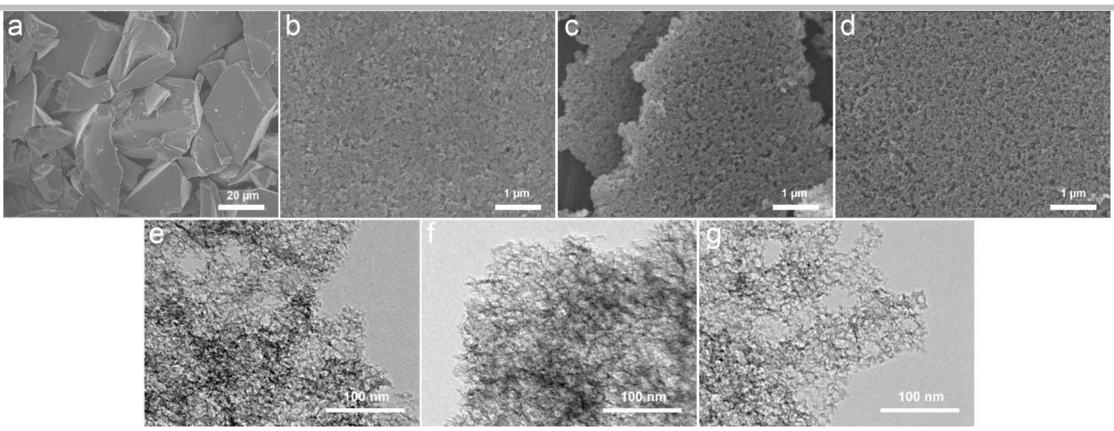

Figure S3. SEM images of (a) PCC_{5-0}, (b) PCC_{5-5}, (c) PCC_{5-7}, and (d) PCC_{5-11}. TEM images of (e) PCC_{5-5}, (f) PCC_{5-7}, and (g) PCC_{5-11}.

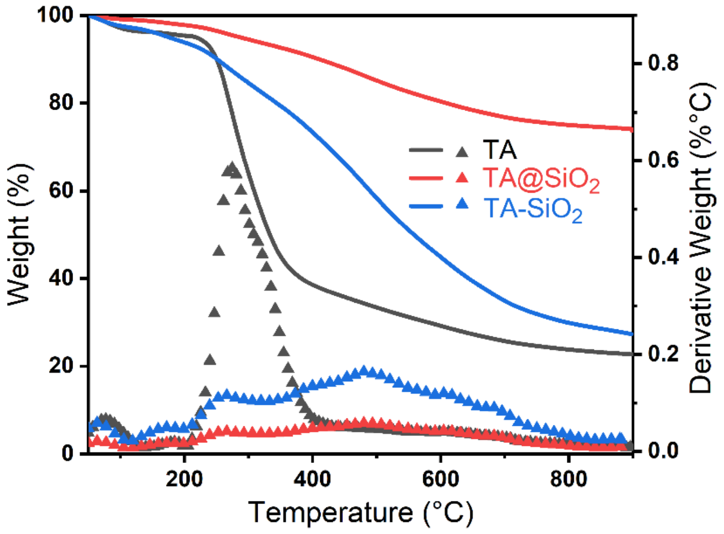

Figure S4. TGA and DTG curves of TA, TA@SiO_2 composites, and TA-SiO_2 (TGA: solid line, DTG: triangular symbol)

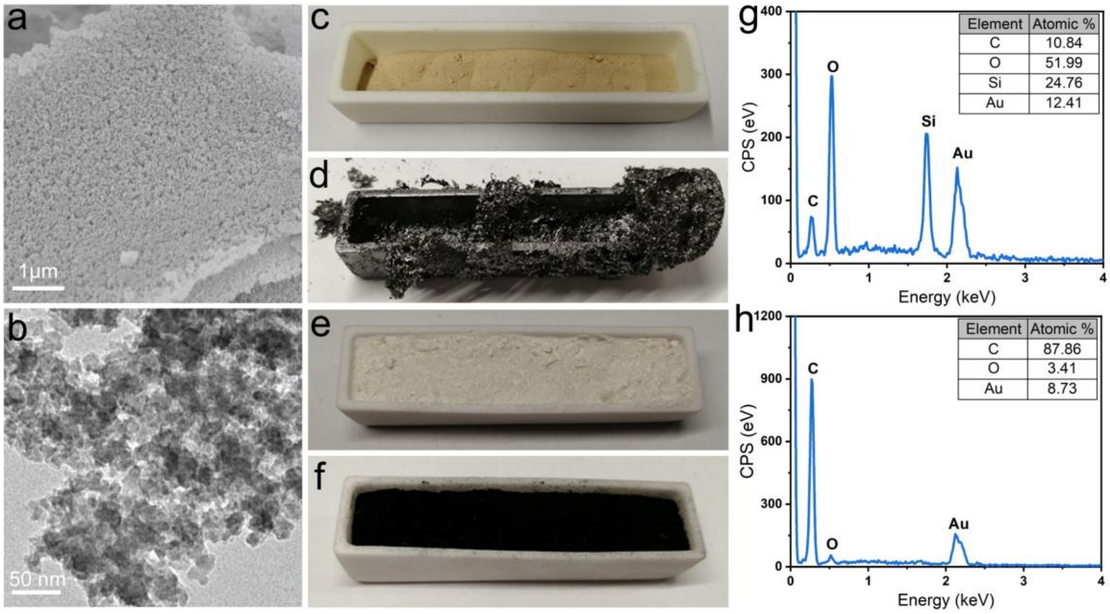

Figure S5. (a) SEM and (b) TEM images of annealed TA@SiO_2 composite. Digital pictures of (c) TA, (d) annealed TA, (e) TA@SiO_2 composite, (f) annealed TA@SiO_2 composite. EDS spectra of (g) annealed TA@SiO_2 composite, and (h) PCC_{900}.

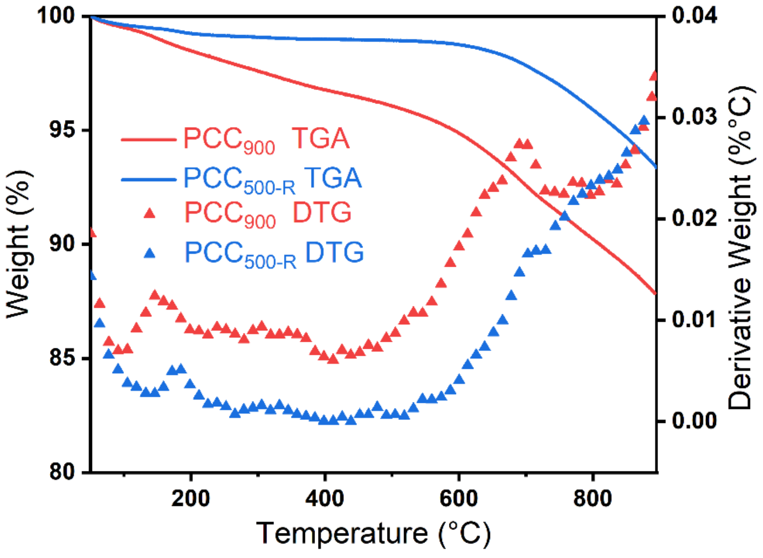

Figure S6. TGA and DTG curves of PCC_{900} and $PCC_{500\text{-}R}$. (TGA: solid line, DTG: triangular symbol)

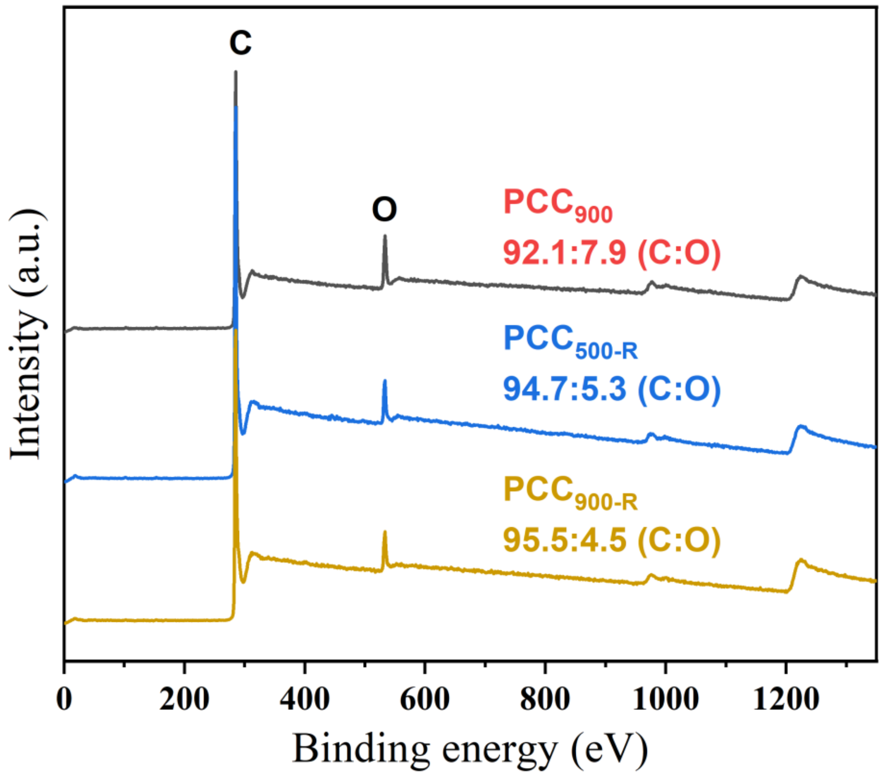

Figure S7. XPS survey scans of PCC_{900}, $PCC_{500\text{-}R}$, and $PCC_{900\text{-}R}$.

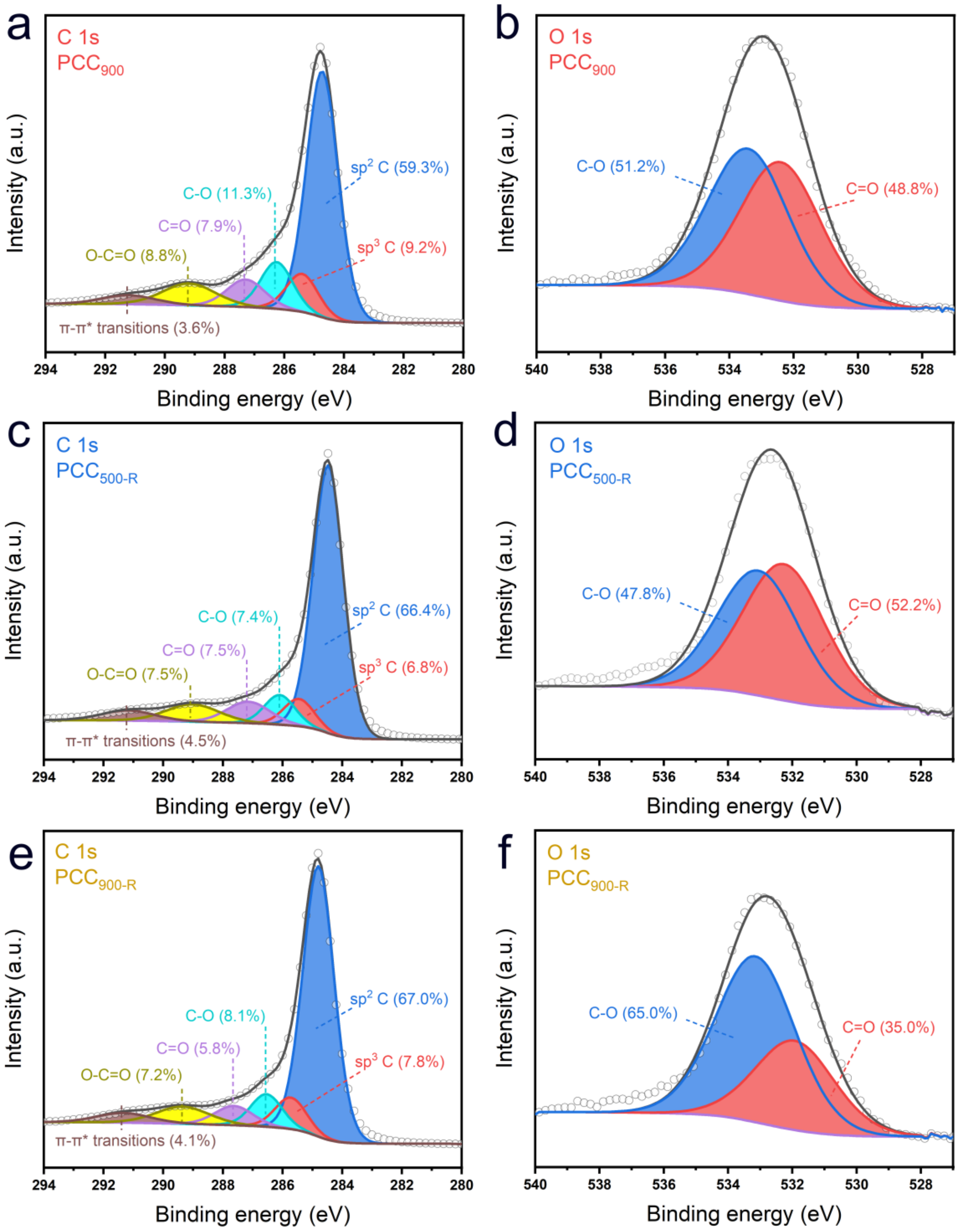

Figure S8. Deconvoluted C 1s XPS spectra of (a) PCC_{900}, (c) PCC_{500-R}, and (e) PCC_{900-R}. Deconvoluted O 1s XPS spectra of (b) PCC_{900}, (d) PCC_{500-R}, and (f) PCC_{900-R}.

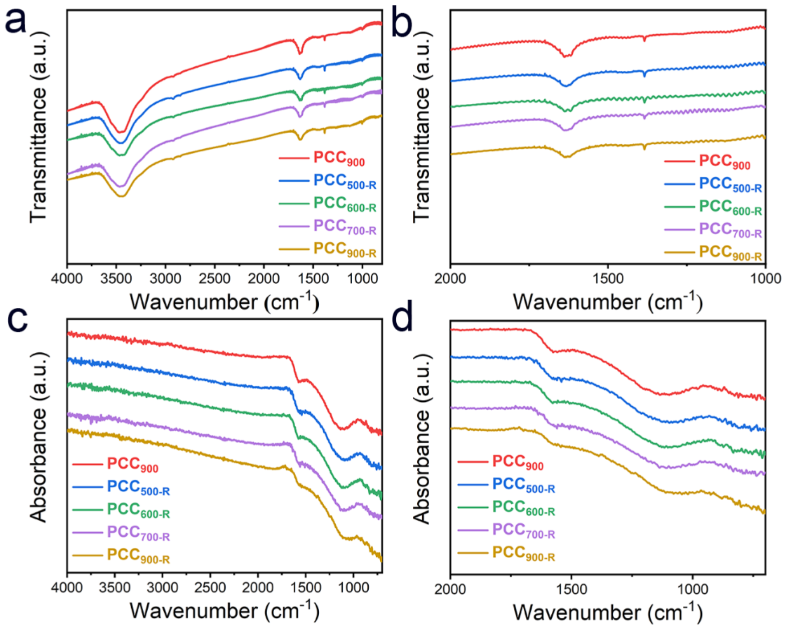

Figure S9. (a-b) ATR-FTIR spectra. (c-d) Transmission-FTIR spectra.

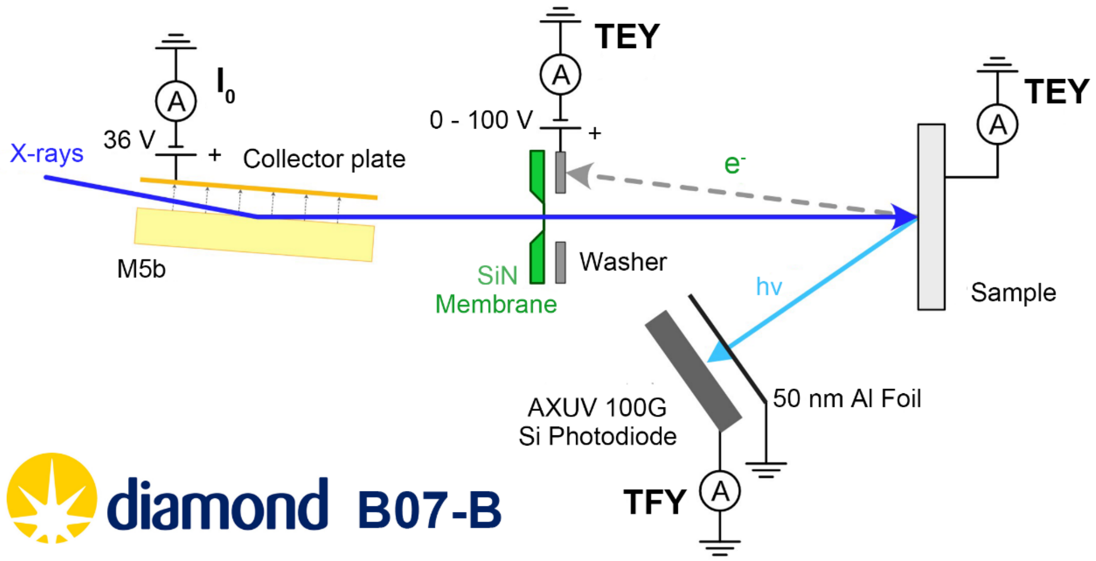

Figure S10. Schematic illustration Beamline end-station set-up of B07-B@Diamond Light Source.

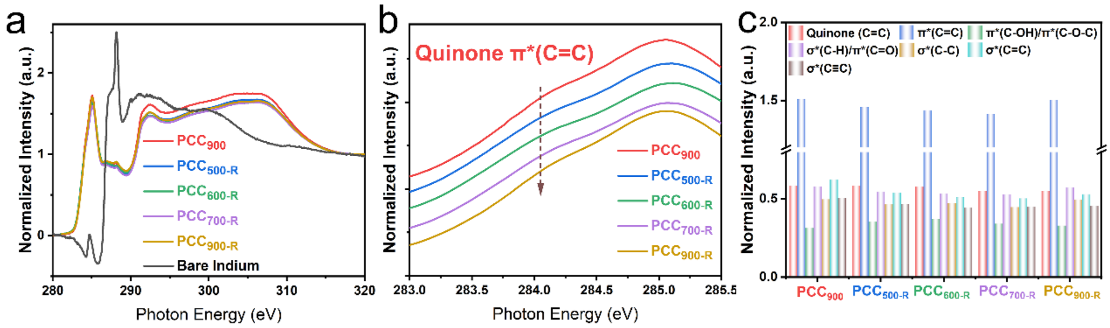

Figure S11. (a) C K-edge NEXAFS spectra. (b) Enlarged C K-edge NEXAF spectra. (c) Normalized intensity contrast of carbon peaks obtained from NEXAFS peak fitting spectra.

Supporting Note 1.

Compared with PCC_{900} and $PCC_{500\text{-}R}$, the intensity of quinone π* (C=C) decreases when PCC_{900} is re-annealed at 600 °C, 700 °C, and 900 °C due to the partial decomposition of the quinone groups. Nevertheless, it is supposed to be noted that the ratio of quinone π* (C=C) peak in the C K-edge NEXAFS spectra is characteristic of the amount of quinone carbon in the whole detected carbon signals. Similarly, the quinone π* (C=O) in the O K-edge NEXAFS spectra is characteristic of the amount of quinone oxygen in the whole detected O signals. In other words, the denominators are different. Considering the oxygen content is less than 8 at% as evidenced by the XPS, the ratio of quinone π* (C=C) in C K-edge is very low. The O K-edge is much more sensitive to determine the change of quinone π* (C=O) for samples. Therefore, for identifying the oxygen functional groups, we prefer to use the O K-edge and the C K-edge is used as complementary.

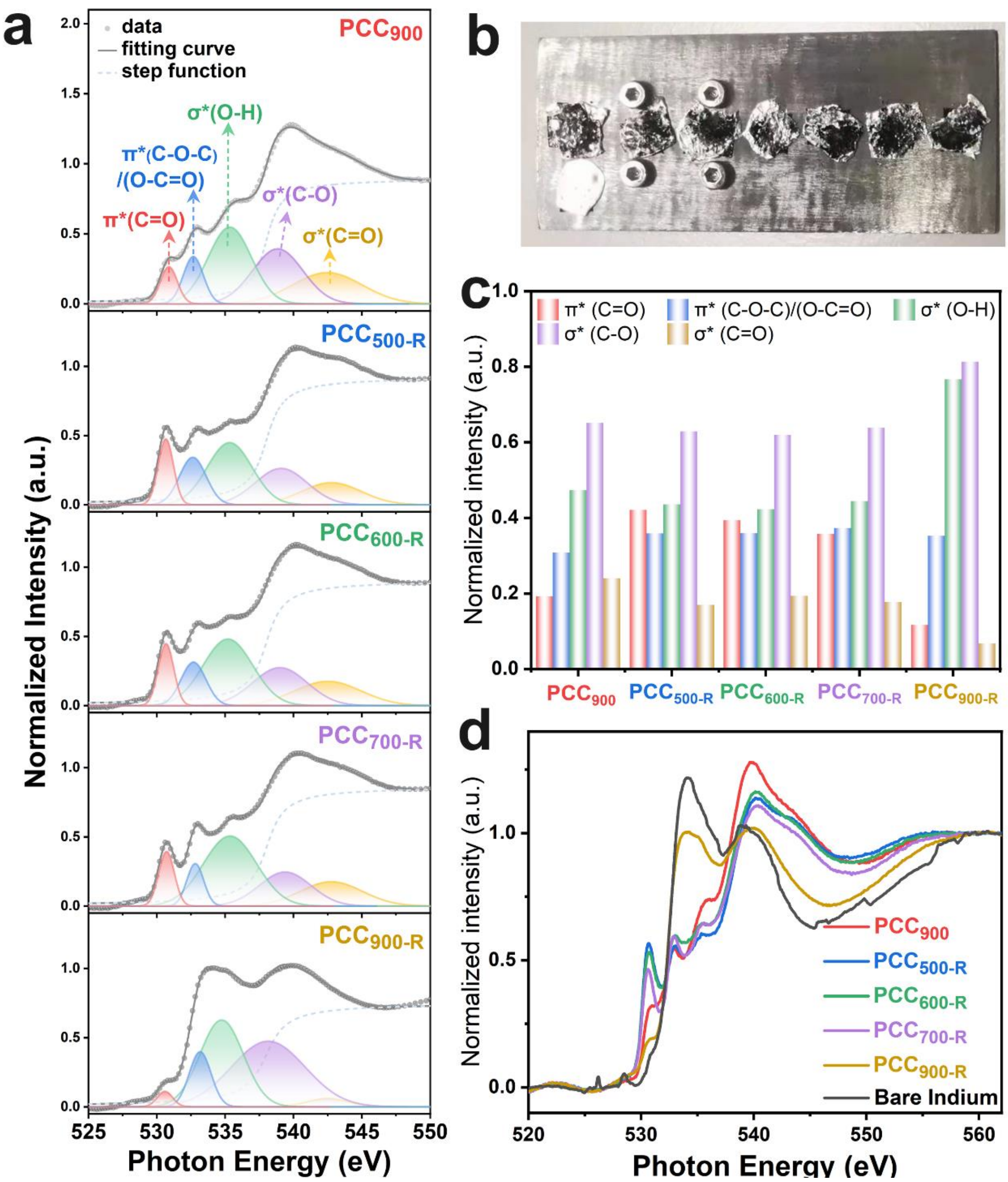

Figure S12. (a) Comparison of O K-edge NEXAFS spectra measured on the indium plate. (b) Digital photos of sample holders with the indium as sample substrates. (c) Normalized intensity contrast of oxygen peaks obtained from NEXAFS peak fitting spectra. (d) Comparison of O K-edge NEXAFS spectra.

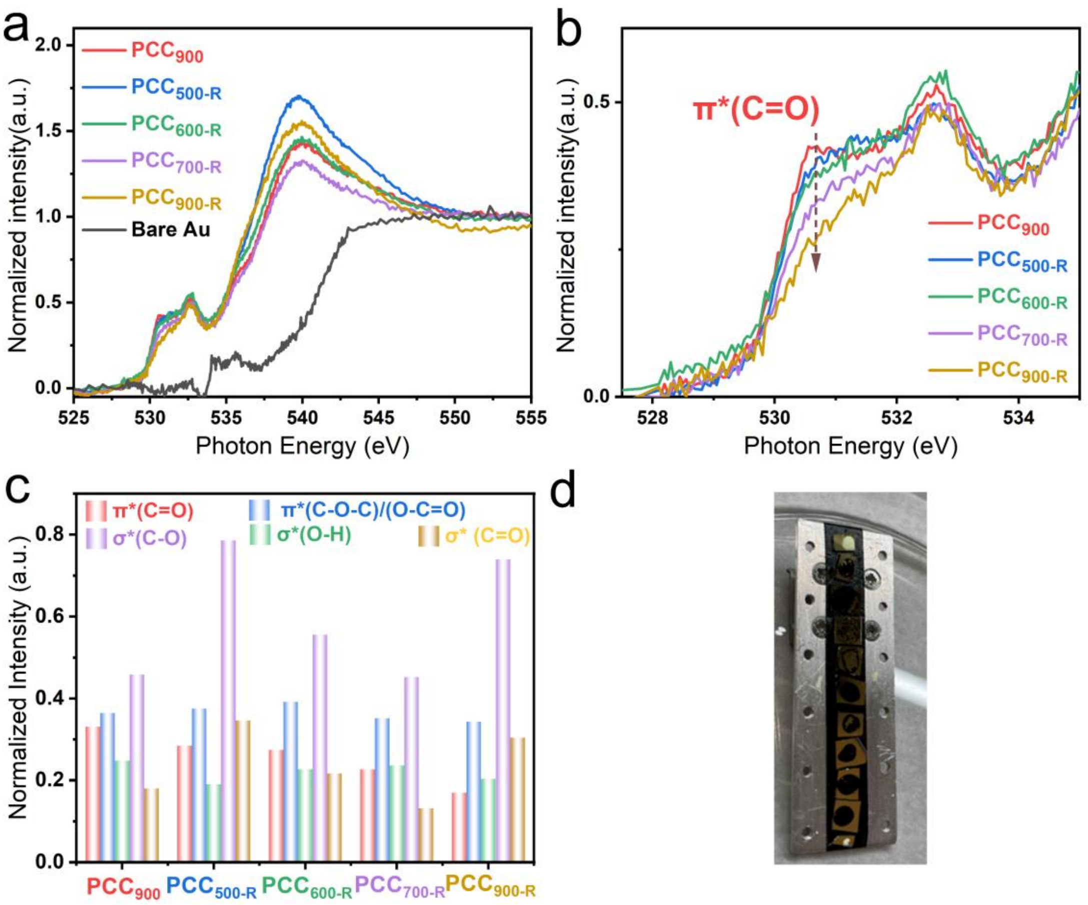

Figure S13. (a) The contrast of O K-edge NEXAFS spectra measured on the Au-coated silicon wafer plate. (b) Enlarged NEXAF spectra. (c) Normalized intensity contrast of oxygen peaks obtained from NEXAFS peak fitting spectra. (d) Digital photos of the sample holder with the Au-coated silicon wafer as the sample substrate.

Supplement Note 2

It is noted that the peak at 539.2 eV shows inconsistency with the re-annealing temperature. This is because the normalization of NEXAFS spectra is performed in the high-energy non-resonant background (555-570 eV) but not at the highest peak position (539.2 eV). Compared with the identification of π* (C=O), the contribution of σ* (C-O) is much more complicated involving the antibonding orbitals of ether, phenol, anhydride, and lactone groups, etc, and the less content of these groups further deepens the uncertainty in determining their contribution. In this case, we mainly depend on π* (C=O) as the indicator to evaluate the variations of quinone groups.

Supporting Note 3.

To exclude the oxygen interference from the substrate, the gold-coated silicon wafer (99.999% (Au) purchased from Sigma-Aldrich (UK) Co., Ltd.) is used. Samples are drop-casted on the cleaned gold substrate using water as the solution and dried on 80 ℃ hot plate for 15 min. A gold substrate is a good option for the measurement of O K-edge NEXAFS spectra due to the instability of gold oxide in the ambient environment. Because of the hydrophobicity of the gold surface, it is relatively easy to drop-cast hydrophobic samples with close surface energy on the gold surface. More attempts are required for samples with much oxygen and hydrophilicity. Alternatively, pressed pellets are also suitable for O K-edge NEXAFS spectra measurement, but it's not feasible for all materials, for example, materials with high hydrophobicity that cannot be easily pressed due to the repellence.

Supporting Note 4.

Generally, the height of the high-energy background in the O K-edge is proportional to the number of oxygen atoms within the information depth of the sample. For PCC_{900} and re-annealed samples, the amount of surface oxygen atoms decreases gradually with elevating the re-annealing temperature, as evidenced by XPS analysis (Figure S 7-8). Therefore, the actual difference in the intensity of π* (C=O) for the samples is supposed to be greater considering the spectra are normalized to a high-energy non-resonant background (555-570 eV).

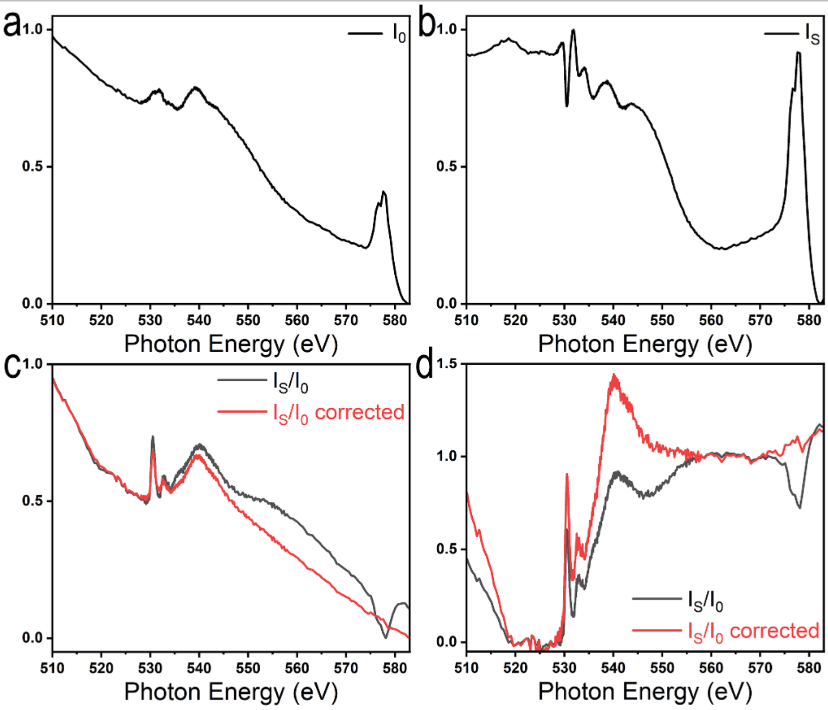

Figure S14. (a) The incident beam intensity (I_0) of O K-edge at B07-B beamtime. (b) Detector current of sample holder (I_S). (c) The comparison of I_S/I_0 and background corrected I_S/I_0. (d) Comparison of normalized I_S/I_0 and corrected I_S/I_0. Taking one scan of PCC_{AAQ} as an example.

Supporting Note 5

Due to the inevitable presence of oxygen species (e.g. oxygen contamination on beamline optics/detectors) and the high sensitivity of beamline detectors, the incident I_0 shows the characteristic feature of oxygen in the range of 525-560 eV (Figure S11a). Normally, the ratio of sample intensity (I_S) to incident beam intensity (I_0) is used as the final NEXAFS signal of samples to remove the additional contribution from I_0. However, the nonlinearity of I_S and I_0 makes it difficult to get rid of the interference of I_0 and present the intrinsic NEXAFS feature of the sample using I_S/I_0. Instead, we tentatively use the equation of $I_S/(a*I_0+b)$ to correct the background. The absence of the unique feature of I_0 in the range of (575-580 eV) is used as the indicator, which is probably caused by chromium (Cr) L_3-edge from Cr binding layers on the mirrors, but it is not supposed to appear in the I_S[13]. As shown in Figure S14c-d, the corrected I_S/I_0 shows no feature from I_0 and a flat background.

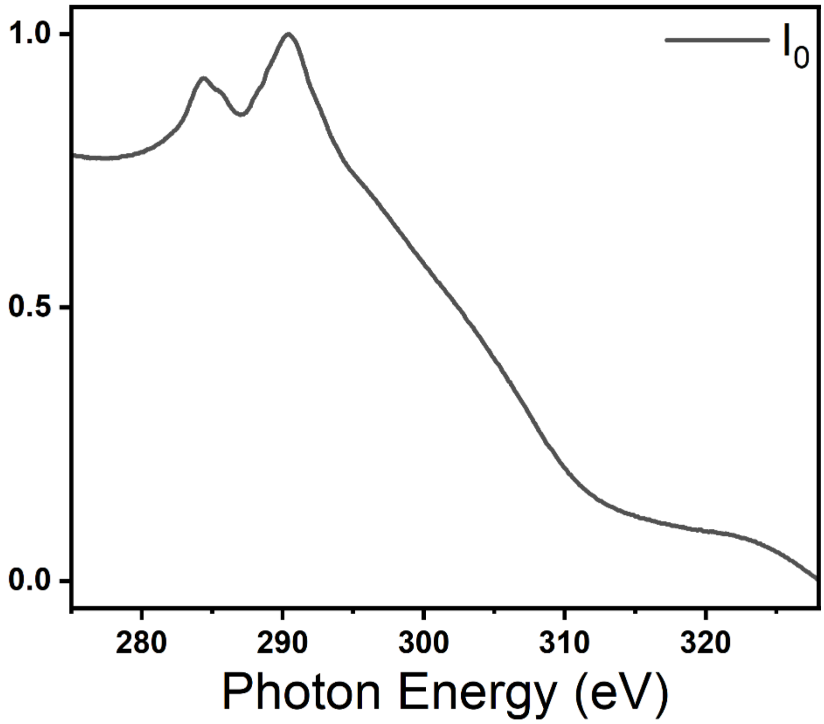

Figure S15. The incident beam current (I_0) of C K-edge

Supporting Note 6

Similarly, the incident beam I_0 of C K-edge shows the feature of carbon in the range of 280-300 eV. However, we do not observe a significant unique feature of I_0 in the post-range of 300-330 eV and C K-edge NEXAFS spectrum does not show much difference for our samples. Therefore, we use I_S/I_0 as the C K-edge NEXAFS data.

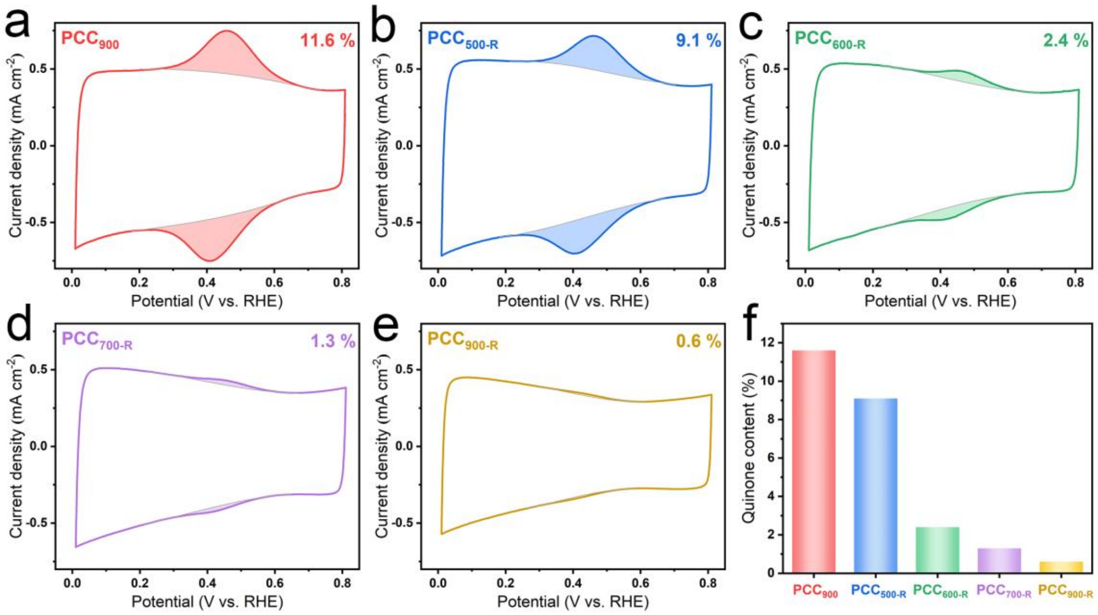

Figure S16. (a-e) The calculations of the quinone content by CV integral method. (f) Quinone content for different samples.

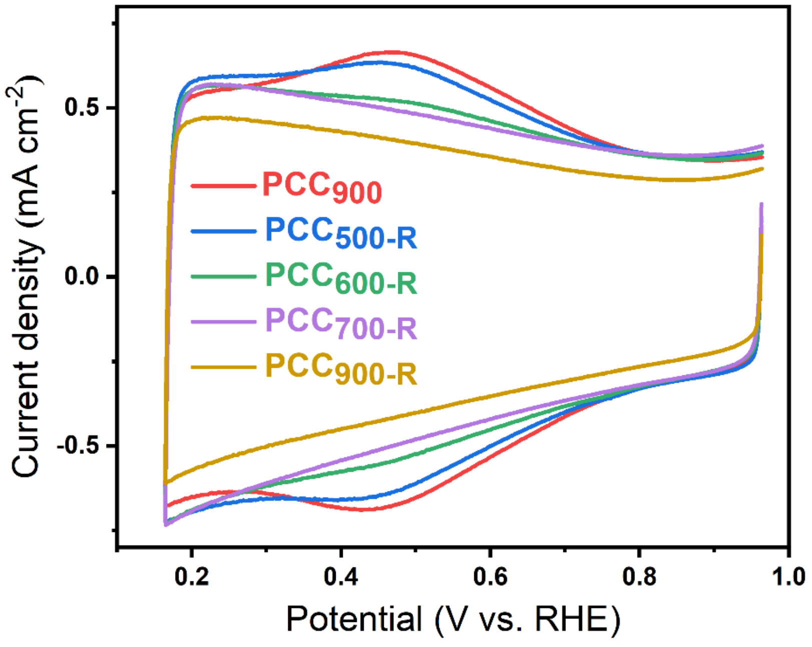

Figure S17. CV curves measured in N_2-saturated 0.1 M KOH with a 50 mV s^{-1} scan rate.

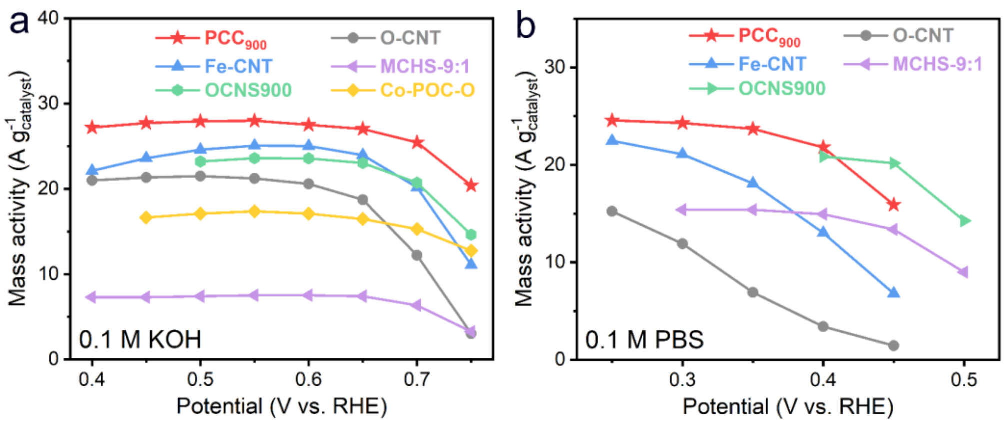

Figure S18. Mass activity contrast in (a) 0.1 M KOH and (b) 0.1 M PBS. The contrast is based on the same loading amount of catalyst on RRDE electrode (0.1 mg cm^{-2}).

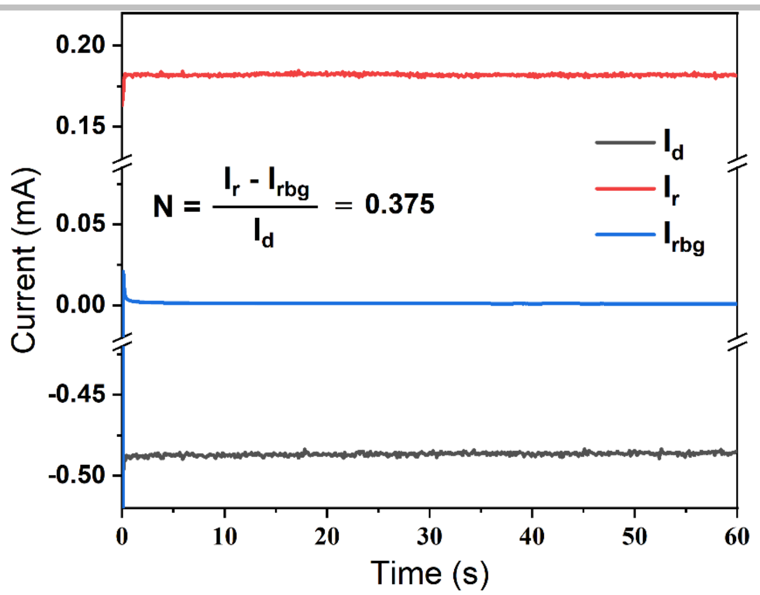

Figure S19. Chronoamperometry curves for a bare RRDE in N_2 saturated 0.1 M KOH and 0.004 M $K_3Fe(CN)_6$ solution.

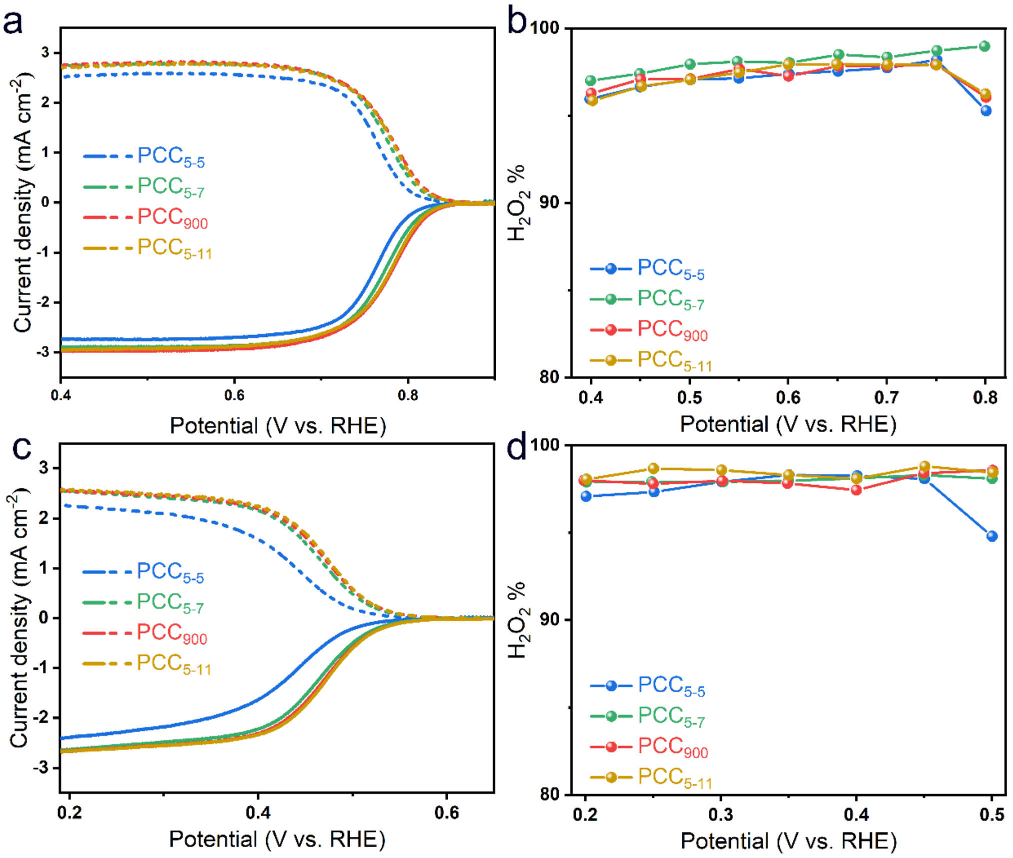

Figure S20. (a) ORR polarization curves of disk current density (solid line) and ring current density (dash line) of PCC_{5-5}, PCC_{5-7}, PCC_{900}, PCC_{5-11} in 0.1 M KOH. (b) Calculated H_2O_2 selectivity (H_2O_2 %). (c) Tafel plots. (d) ORR polarization curves in 0.1 M PBS (pH = 7). (e) Calculated H_2O_2 selectivity (H_2O_2 %).

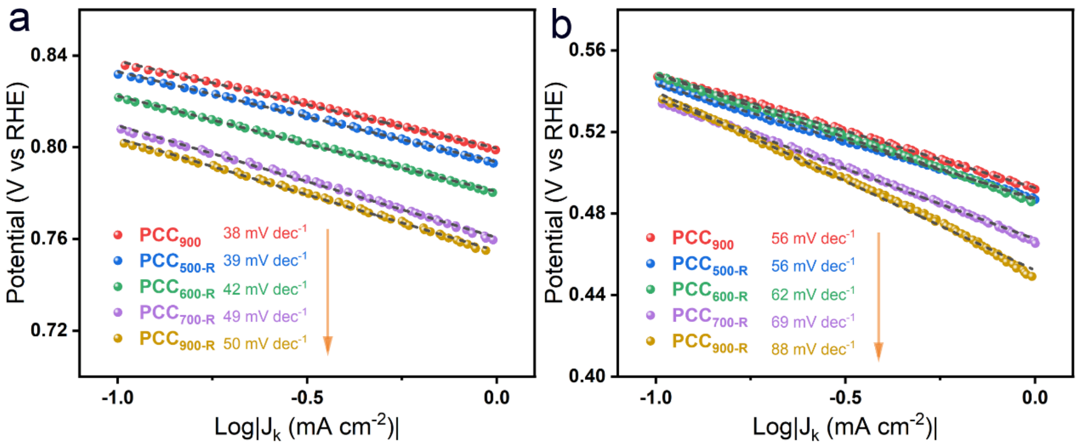

Figure S21. Tafel plots in (a) 0.1 M KOH and (b) 0.1 M PBS.

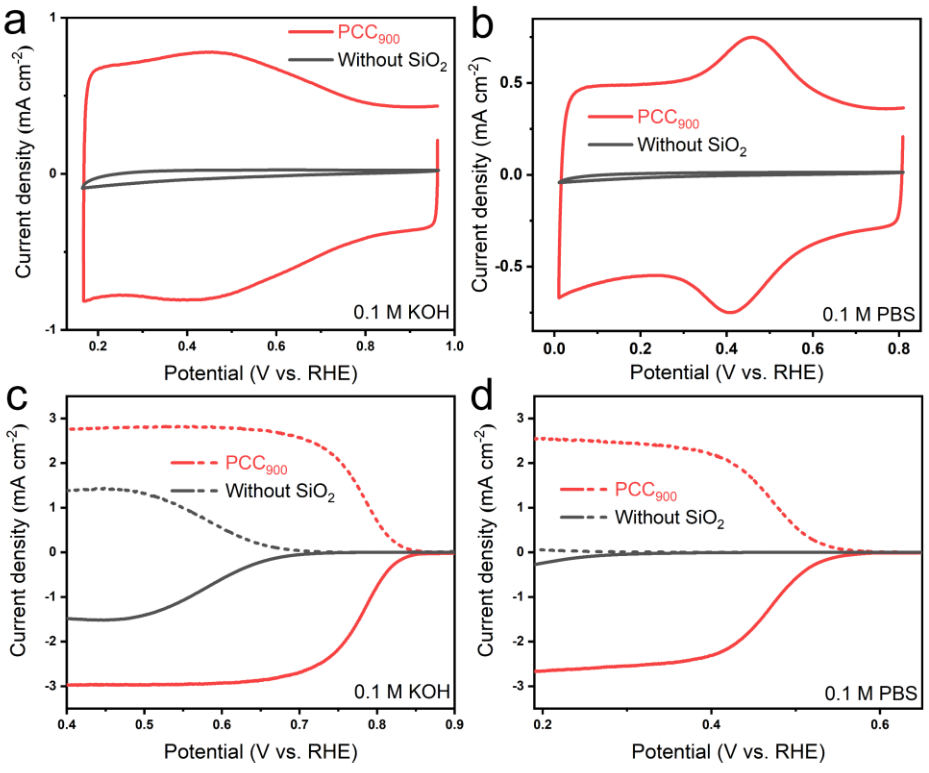

Figure S22. CV curves of TA (without SiO_2) and PCC_{900} in N_2-saturated 0.1 M KOH (a) and 0.1 M PBS (b). ORR polarization curves in TA (without SiO_2) and PCC_{900} in 0.1 M KOH (c) and 0.1 M PBS (d).

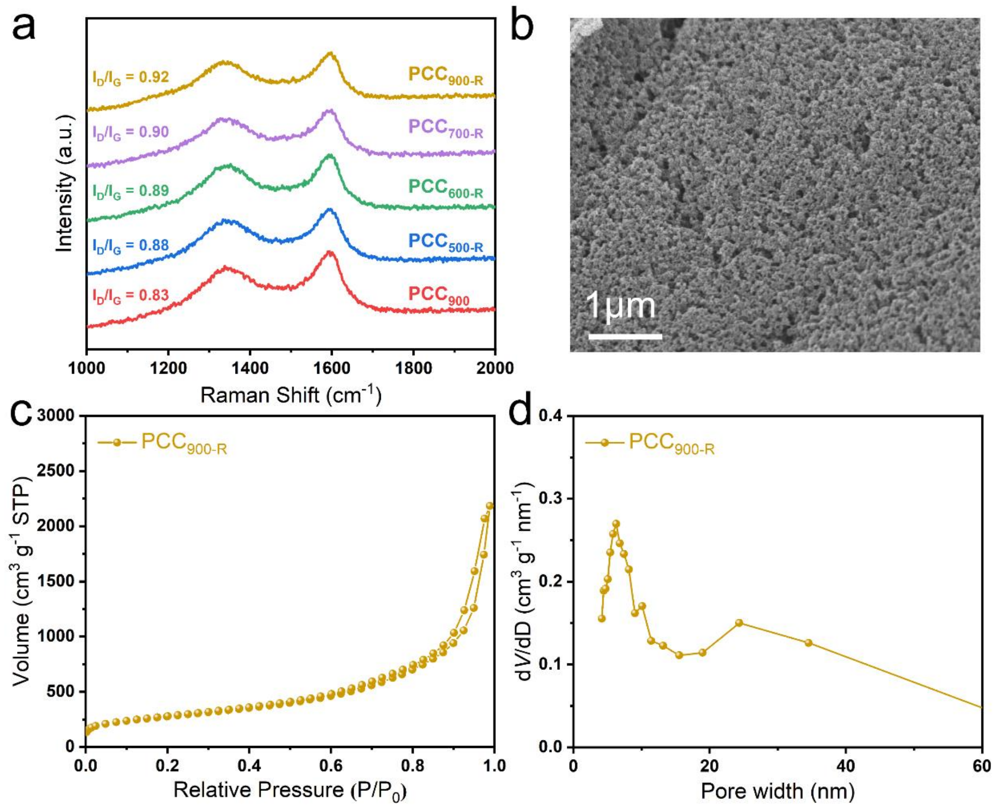

Figure S23. (a) Raman spectra. (b) SEM image, (c) N_2 adsorption/desorption isotherms, and (d) BJH pore size distribution of PCC_{900-R}.

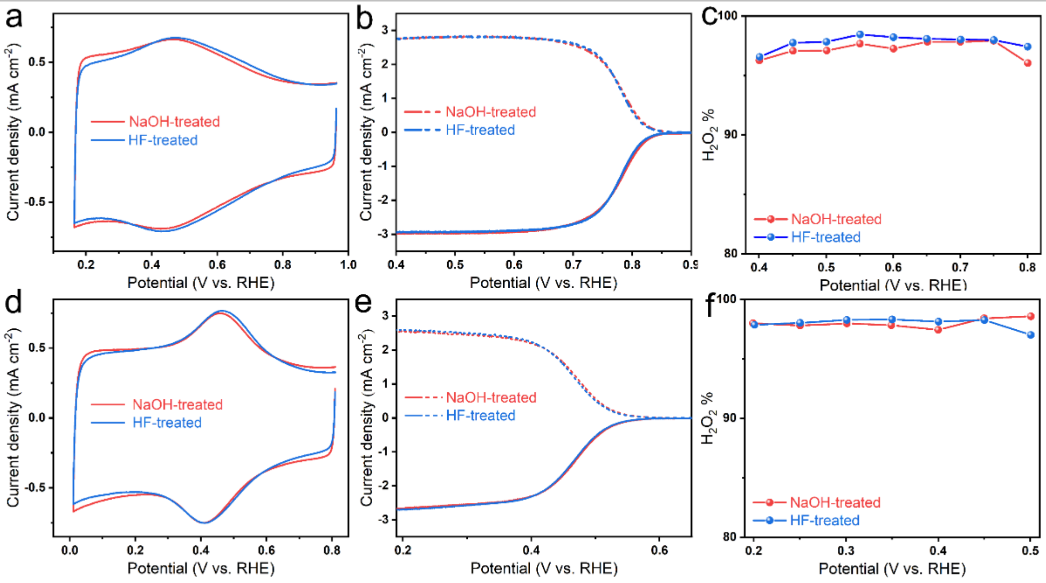

Figure S24. (a) CV curves of NaOH-treated and HF-treated PCC_{900} in N_2-saturated 0.1 M KOH. (b) ORR polarization curves in 0.1 M KOH. (c) Calculated H_2O_2 selectivity (H_2O_2 %). (d) CV curves in N_2-saturated 0.1 M PBS. (e) ORR polarization curves in 0.1 M PBS. (f) Calculated H_2O_2 selectivity (H_2O_2 %).

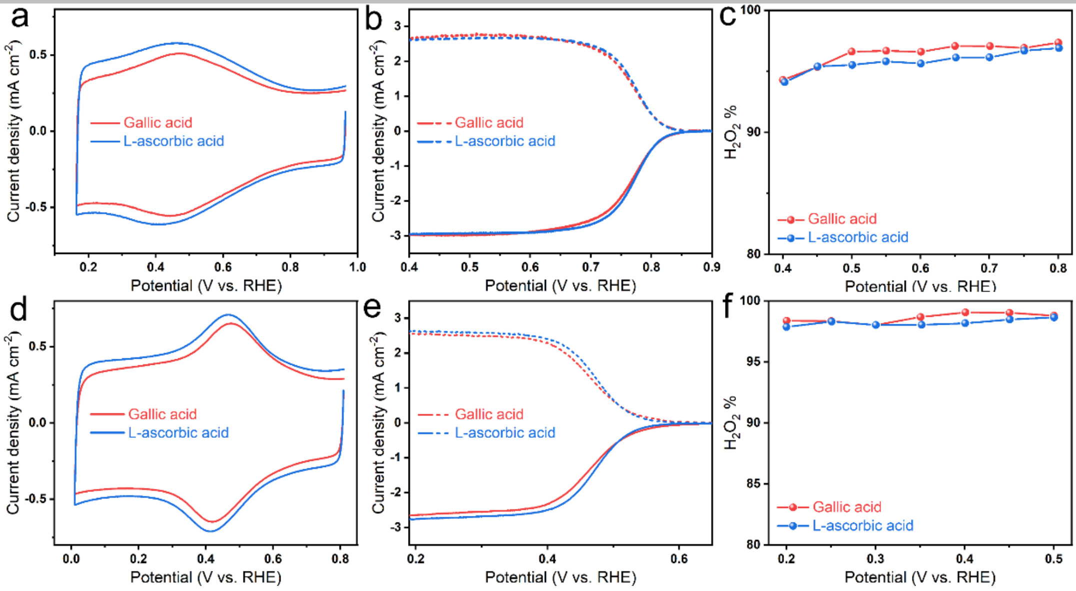

Figure S25. (a) CV curves of PCC_{900} using gallic acid and L-ascorbic acid as carbon precursors in N_2-saturated 0.1 M KOH. (b) ORR polarization curves in 0.1 M KOH. (c) Calculated H_2O_2 selectivity (H_2O_2 %). (d) CV curves in N_2-saturated 0.1 M PBS. (e) ORR polarization curves in 0.1 M PBS. (f) Calculated H_2O_2 selectivity (H_2O_2 %).

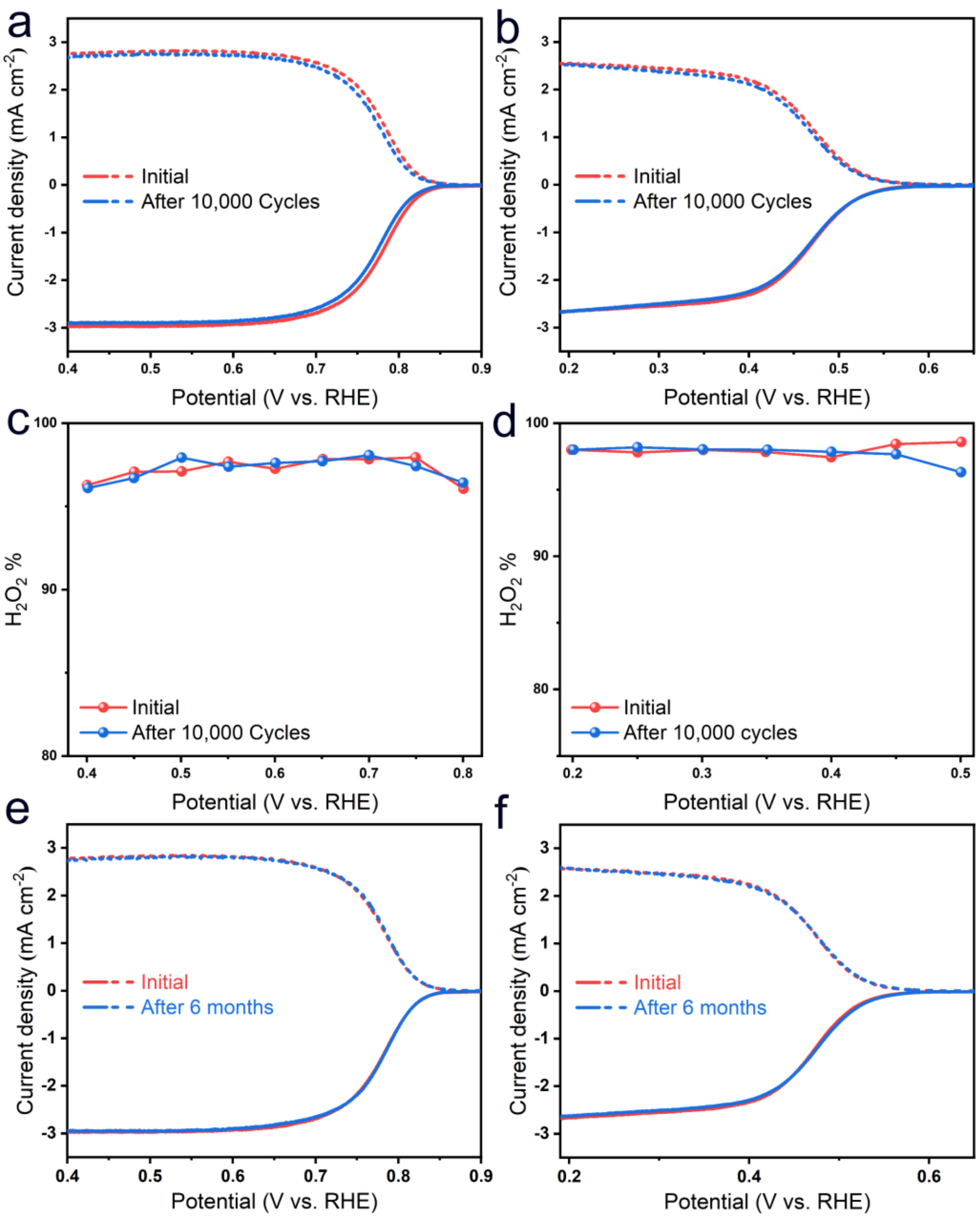

Figure S26. ORR polarization curves in (a) 0.1 M KOH and (b) 0.1 M PBS. Calculated H_2O_2 selectivity (H_2O_2 %) in (c) 0.1 M KOH and (d) 0.1 M PBS. ORR polarization curves of initial and aged PCC_{900} in (e) 0.1 M KOH and (f) 0.1 M PBS.

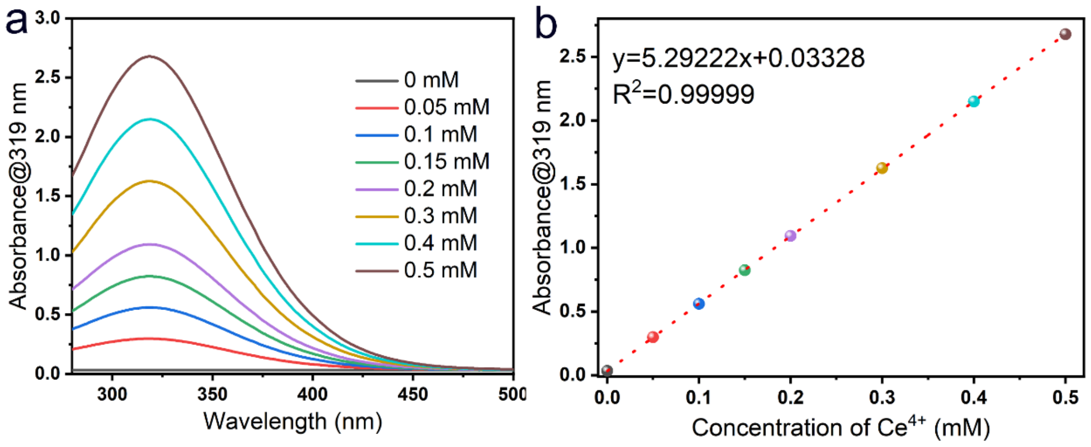

Figure S27. (a) Absorbance spectra of standard Ce(SO4)2 solutions (up to 0.5 mM) in 0.5 M H_2SO_4, generating a linear calibration curve (shown as an inset) at the peak wavelength (319 nm).

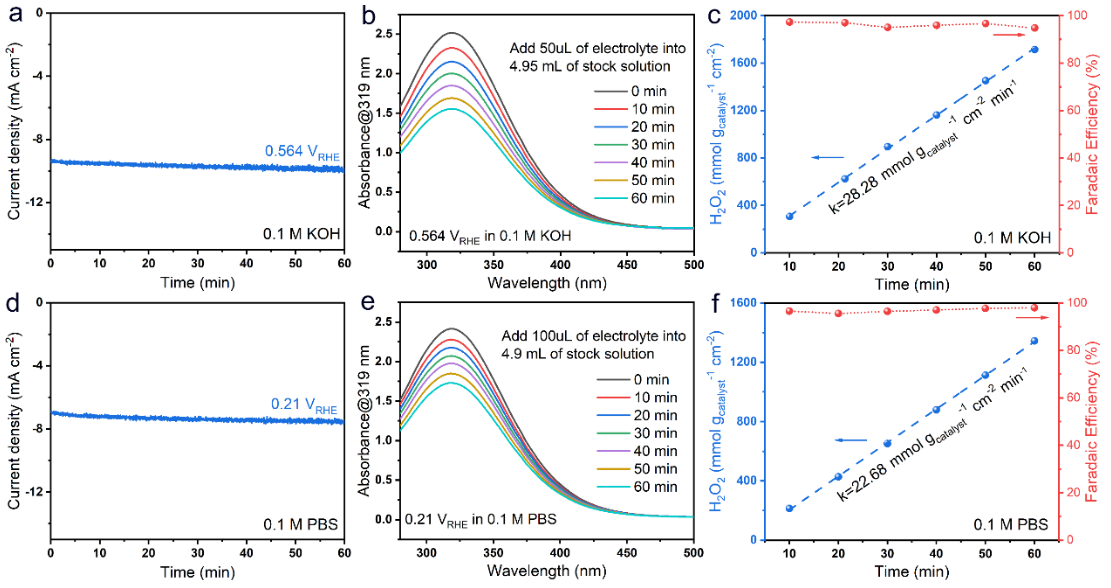

Figure S28. (a) Chronoamperometry curve at 0.564 V_{RHE} (-0.4V vs. Ag/AgCl) in 0.1 M KOH. (b) UV-vis spectra of Ce^{4+} solutions after reacting with electrolytes at different times. (c) Produced H_2O_2 concentration and related Faradaic efficiency in 0.1 M KOH for PCC_{900}. (d) Chronoamperometry curve at 0.21 V_{RHE} (-0.4V vs. Ag/AgCl) in 0.1 M PBS. (e) UV-vis spectra of Ce^{4+} solutions after reacting with electrolytes at different times. (f) Produced H_2O_2 concentration and related Faradaic efficiency in 0.1 M PBS for PCC_{900}.

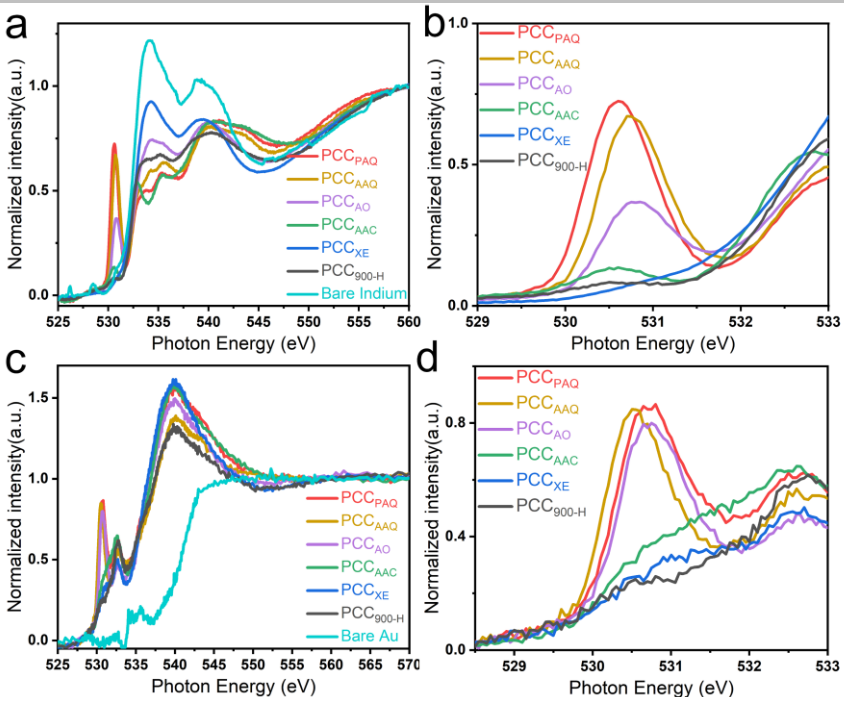

Figure S29. (a) O K-edge NEXAFS spectra measured of sample immobilised on indium plate. (b) Enlarged area of corresponding NEXAFS spectra in figure a. (c) O K-edge NEXAFS spectra measured of sample immobilised on the gold-coated silicon wafer. (d) Enlarged area of corresponding NEXAFS spectra in figure c.

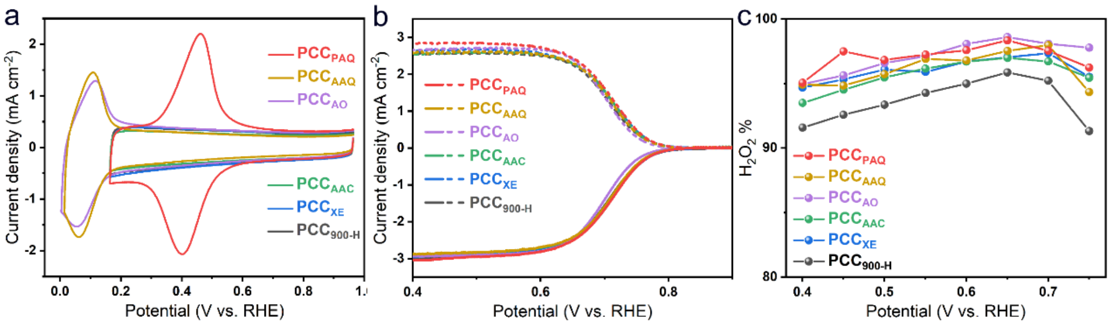

Figure S30. (a) CV curves in N_2-saturated 0.1 M KOH. (b) ORR polarization curves of disk current density (solid line) and ring current density (dash line) in 0.1 M KOH. (c) Calculated H_2O_2 selectivity (H_2O_2 %).

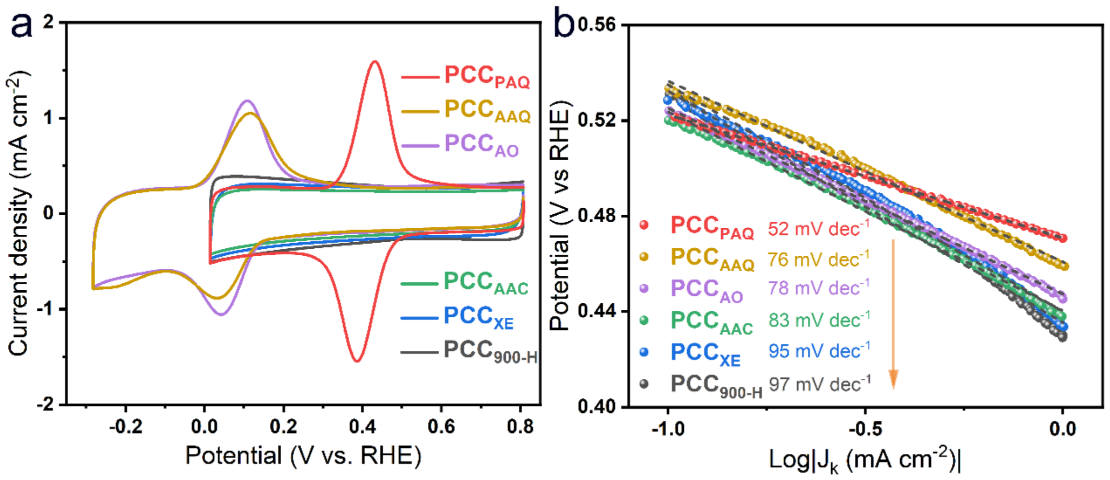

Figure S31. (a) CV curves in N_2-saturated 0.1 M PBS. (b) Tafel plots.

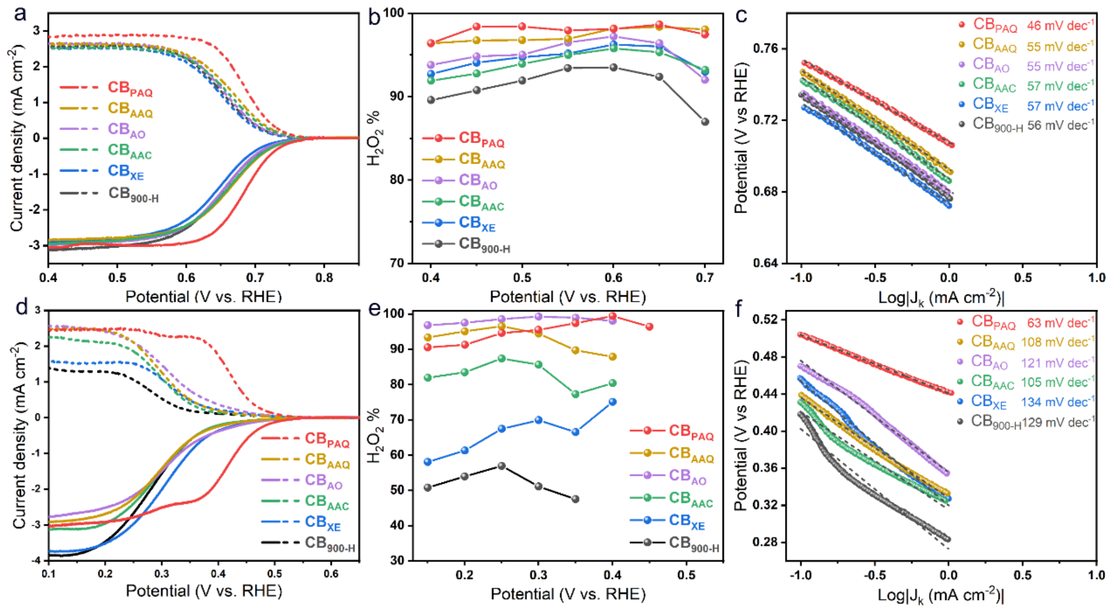

Figure S32. (a) ORR polarization curves of disk current density (solid line) and ring current density (dash line) of $CB_{900\text{-}H}$, CB_{XE}, CB_{AAC}, CB_{AO}, CB_{AAQ}, and CB_{PAQ} in 0.1 M KOH. (b) Calculated H_2O_2 selectivity (H_2O_2 %). (c) Tafel plots. (d) ORR polarization curves in 0.1 M PBS (pH = 7). (e) Calculated H_2O_2 selectivity (H_2O_2 %). (f) Tafel plots.

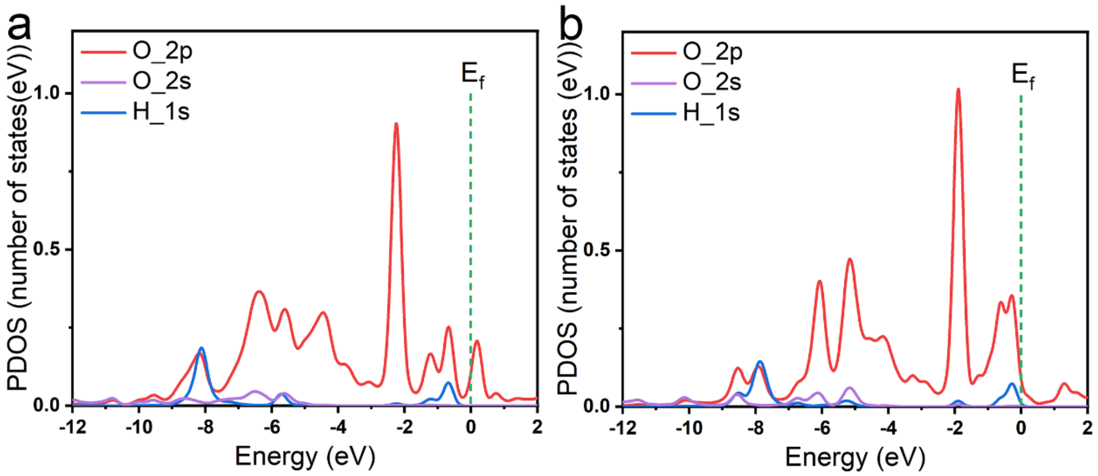

Figure S33. (a) The partial density of states for (a) $C_{together}$ and (b) $Z_{separate}$ systems. The Fermi energy is indicated by the dashed line passing through 0 eV. The red, violet and blue lines indicate O 2p, O 2s, and H 1s states.

Table S1. Specific surface area from BET data.

Sample	BET surface area (m^2/g)	Pore volume (cm^3/g)
$PCC_{5\text{-}5}$	1081.61	2.70
$PCC_{5\text{-}7}$	1167.91	3.76
PCC_{900}	1466.92	3.67
$PCC_{5\text{-}11}$	1456.92	3.45
$PCC_{900\text{-}R}$	1165.65	3.73

Table S2. The 2e$^-$ ORR performance comparison of PCC_{900} and reported catalysts in 0.1 M KOH.

Catalyst	Loading amount (mg cm^{-2})	Potential @ 0.1 mA cm^{-2} (V)	Max. H_2O_2 Selectivity (%)	Potential @ 1 mA cm^{-2} (V)	H_2O_2 Selectivity at 0.7 V (%)	Reference
PCC_{900}	0.1	0.83	97.9	0.79	97.8	This work
O-CNT	0.1	0.75	89	0.72	~90.0	[14]
Fe-O-CNT	0.1	0.822	95	0.74	~95	[15]
O-C(Al)	0.08	0.824	97	0.76	~97	[16]
Co_1-NG(O)	0.01	0.8	80	0.745	~76	[17]
Co-POC-O	0.1	0.84	84	0.78	~84	[18]
F-mrGO	0.01	0.78	>95	0.68	>95	[19]
MCHS-9:1	0.1	0.83	54.7	0.77	~58	[20]
GNPC=O,1	N.A.	0.815	97.8	0.77	~97	[3]
BN-C1	0.51	0.82	90	0.76	~88	[21]
AC-CO_2B	N.A.	0.83	90	0.77	~83	[22]
g-N-CNH	0.36	0.71	60	0.63	~80	[23]
$OCNS_{900}$	0.1	0.825	~91	0.78	~90	[24]
O-GOMC	0.05	~0.8	~90	0.75	~90	[25]

Table S3. The 2e[-] ORR performance comparison of PCC_{900} and reported catalysts in neutral media.

Catalyst	Electrolyte	Loading amount (mg cm^{-2})	Potential @0.1 mA cm^{-2} (V)	Max. H_2O_2 Selectivity (%)	Potential @1 mA cm^{-2} (V)	H_2O_2 Selectivity at 0.4 V(%)	Reference
PCC_{900}	0.1 M PBS (pH 7)	0.1	0.55	98.5	0.48	97.43	This work
O-CNT	0.1 M PBS (pH 7)	0.1	0.43	80	0.3	~85	[14]
Fe-O-CNT	0.1 M PBS (pH 7.2)	0.1	0.48	90	0.42	~90	[15]
O-C(Al)	0.1 M PBS (pH 7)	0.08	0.526	90	0.4	~88	[16]
Co_1-NG(O)	0.1 M PBS (pH 7.4)	0.01	0.59	69	0.38	~65	[17]
PEI50CMK3	0.1 M K_2SO_4 (pH 7)	0.05	0.39	88	0.27	~88	[26]
MCHS-9:1	0.1 M PBS (pH 8)	0.1	0.57	99	~0.51	~91	[20]
Co-N-C	0.1 M K_2SO_4 (pH 7)	0.1	0.45	59	0.4	~60	[27]
$OCNS_{900}$	0.1 M PBS (pH 7)	0.1	~0.58	94	~0.51	~85	[24]
O-GOMC	0.1 M PBS (pH N.A.)	0.05	~0.53	93	~0.48	~85	[25]

Table S4. The H_2O_2 production performance comparison of PCC_{900} and reported catalysts.

Catalyst	Electrolyte	Loading amount (mg cm^{-2})	Loading area (cm^2)	Method	H_2O_2 yield (mmol g^{-1} h^{-1})	FE (%)	Reference
PCC_{900}	0.1 M KOH (20 mL)	0.1	1	H-cell -0.4 $V_{Ag/AgCl}$	1697	>90 %	This work
PCC_{900}	0.1 M PBS (20 mL)	0.1	1	H-cell -0.4 $V_{Ag/AgCl}$	1361	>90 %	This work
O-CNT	1 M KOH (25 mL)	~2	N.A.	H-cell 50 mA	~1452 (~1975mg/L in 0.5 h)	>90 %	[14]
Fe-O-CNT	1 M KOH (N.A.)	0.5	2.5	Fuel cell 43 mA cm^{-2}	~1600	95.40%	[15]
Co_1-NG(O)	0.1 M KOH (15 mL)	1	1	H-cell 50 mA	~418	N.A.	[17]
Co-POC-O	0.1 M KOH (50mL)	0.25	10	Single cell 100 mA	~478 (813 mg/L/h)	>60 %	[18]
$OCNS_{900}$	0.1 M KOH (N.A.)	1	1	Fuel Cell 50 mA cm^{-2}	770	>80 %	
Co-N-C	0.1 M KOH (N.A.)	0.1	10	Flow cell 500 mA	4330	~60%	[27]
Co-N-C	0.1 M KOH (N.A.)	N.A.	N.A.	H-cell 0.1 V_{RHE}	193.1	~50%	[27]
Co-N-C	0.1 M K_2SO_4 (N.A.)	N.A.	N.A.	H-cell 0.1 V_{RHE}	89.8	~40%	[27]
PEI50CMK3	0.1 M KOH (N.A.)	N.A.	N.A.	H-cell 0.1 V_{RHE}	457.3	>50%	[26]
PEI50CMK3	0.1 M K_2SO_4 (N.A.)	N.A.	N.A.	H-cell 0.2 V_{RHE}	570.1	>50%	[26]
O-C(Al)	0.5 M Na_2SO_4 (20 mL)	1	N.A.	H-cell 30 mA cm^{-2}	~510 (867ppm in 1 h)	>90%	[16]

Table S5. ZPE, G_{DFT} and TS for free energies.

Model	ZPE (eV)	G_{DFT} (eV)	TS (eV)
H_2	0.29	-6.76	0.41
H_2O	0.60	-14.23	0.59
H_2O_2	0.71	-18.15	0.72
OOH*	0.39	-13.26	0.18

* Adsorbed species

References

[1] B. Ravel, M. Newville, *J. Synchrotron Radiat.* **2005**, *12*, 537-541.

[2] R. F. Zhou, Y. Zheng, M. Jaroniec, S. Z. Qiao, *ACS Catal.* **2016**, *6*, 4720-4728.

[3] G. F. Han, F. Li, W. Zou, M. Karamad, J. P. Jeon, S. W. Kim, S. J. Kim, Y. Bu, Z. Fu, Y. Lu, S. Siahrostami, J. B. Baek, *Nat. Commun.* **2020**, *11*, 2209.

[4] G. Kresse, J. Furthmüller, *Phys. Rev. B* **1996**, *54*, 11169.

[5] P. E. Blöchl, *Phys. Rev. B* **1994**, *50*, 17953.

[6] J. P. Perdew, K. Burke, M. Ernzerhof, *Phys. Rev. Lett.* **1996**, *77*, 3865.

[7] S. Grimme, J. Antony, S. Ehrlich, H. Krieg, *J. Chem. Phys.* **2010**, *132*, 154104.

[8] a) M. Yin, M. L. Cohen, *Phys. Rev. B* **1984**, *29*, 6996; b) L. Sahoo, S. Mondal, A. Gloskovskii, A. Chutia, U. K. Gautam, *J. Mater. Chem A* **2021**, *9*, 10966-10978.

[9] G. Makov, M. Payne, *Phys. Rev. B* **1995**, *51*, 4014.

[10] a) E. Sanville, S. D. Kenny, R. Smith, G. Henkelman, *J. Comput. Chem.* **2007**, *28*, 899-908; b) W. Tang, E. Sanville, G. Henkelman, *J. Phys.: Condens. Matter* **2009**, *21*, 084204.

[11] J. K. Nørskov, J. Rossmeisl, A. Logadottir, L. Lindqvist, J. R. Kitchin, T. Bligaard, H. Jónsson, *J. Phys. Chem. B* **2004**, *108*, 17886-17892.

[12] J. J. Gao, H. B. Yang, X. Huang, S. F. Hung, W. Z. Cai, C. M. Jia, S. Miao, H. M. Chen, X. F. Yang, Y. Q. Huang, T. Zhang, B. Liu, *Chem* **2020**, *6*, 658-674.

[13] G. Held, F. Venturini, D. C. Grinter, P. Ferrer, R. Arrigo, L. Deacon, W. Q. Garzon, K. Roy, A. Large, C. Stephens, A. Watts, P. Larkin, M. Hand, H. C. Wang, L. Pratt, J. J. Mudd, T. Richardson, S. Patel, M. Hillman, S. Scott, *J.Synchrotron Radiat.* **2020**, *27*, 1153-1166.

[14] Z. Y. Lu, G. X. Chen, S. Siahrostami, Z. H. Chen, K. Liu, J. Xie, L. Liao, T. Wu, D. C. Lin, Y. Y. Liu, T. F. Jaramillo, J. K. Norskov, Y. Cui, *Nat. Catal.* **2018**, *1*, 156-162.

[15] K. Jiang, S. Back, A. J. Akey, C. Xia, Y. Hu, W. Liang, D. Schaak, E. Stavitski, J. K. Norskov, S. Siahrostami, H. Wang, *Nat. Commun.* **2019**, *10*, 3997.

[16] Q. Yang, W. Xu, S. Gong, G. Zheng, Z. Tian, Y. Wen, L. Peng, L. Zhang, Z. Lu, L. Chen, *Nat. Commun.* **2020**, *11*, 5478.

[17] E. Jung, H. Shin, B.-H. Lee, V. Efremov, S. Lee, H. S. Lee, J. Kim, W. H. Antink, S. Park, K.-S. Lee, *Nat. Mater.* **2020**, *19*, 436-442.

[18] B. Q. Li, C. X. Zhao, J. N. Liu, Q. Zhang, *Adv. Mater.* **2019**, *31*, 1808173.

[19] H. W. Kim, M. B. Ross, N. Kornienko, L. Zhang, J. H. Guo, P. D. Yang, B. D. McCloskey, *Nat. Catal.* **2018**, *1*, 282-290.

[20] Y. Y. Pang, K. Wang, H. Xie, Y. Sun, M. M. Titirici, G. L. Chai, *ACS Catal.* **2020**, *10*, 7434-7442.

[21] S. Chen, Z. Chen, S. Siahrostami, D. Higgins, D. Nordlund, D. Sokaras, T. R. Kim, Y. Liu, X. Yan, E. Nilsson, R. Sinclair, J. K. Norskov, T. F. Jaramillo, Z. Bao, *J. Am. Chem. Soc.* **2018**, *140*, 7851-7859.

[22] F. Sun, C. W. Yang, Z. B. Qu, W. Zhou, Y. N. Ding, J. H. Gao, G. B. Zhao, D. F. Xing, Y. F. Lu, *Appl. Catal. B Environ.* **2021**, *286*, 119860.

[23] D. Iglesias, A. Giuliani, M. Melchionna, S. Marchesan, A. Criado, L. Nasi, M. Bevilacqua, C. Tavagnacco, F. Vizza, M. Prato, *Chem* **2018**, *4*, 106-123.

[24] S. Chen, T. Luo, K. Chen, Y. Lin, J. Fu, K. Liu, C. Cai, Q. Wang, H. Li, X. Li, J. Hu, H. Li, M. Zhu, M. Liu, *Angew. Chem. Int. Ed.* **2021**, *60*, 16607-16614.

[25] J. S. Lim, J. H. Kim, J. Woo, D. Baek, K. Ihm, T. J. Shin, Y. J. Sa, S. H. Joo, *Chem* **2021**, *7*, 3114-3130.

[26] Y. Sun, S. Li, Z. P. Jovanov, D. Bernsmeier, H. Wang, B. Paul, X. Wang, S. Kühl, P. Strasser, *ChemSusChem* **2018**, *11*, 3388-3395.

[27] Y. Sun, L. Silvioli, N. R. Sahraie, W. Ju, J. Li, A. Zitolo, S. Li, A. Bagger, L. Arnarson, X. Wang, *J. Am. Chem. Soc.* **2019**, *141*, 12372-12381.

Author contributions

L. L. and G. H. conceived the project and designed the experiments; L. L. synthesized and characterized the materials; L. L., L. K., P. F. and D. G performed NEXAFS measurement and analysis; A. C. and J. F. performed DFT calculations; M. M. performed the HF treatment work; Y. T., F. Z., and F. G. performed data analysis; L. L., L. K., D. G. H., and C. A. performed STEM measurements; Y. H. performed the freeze-dying treatment; J. G. performed nitrogen adsorption–desorption isotherms measurement; I. P., S. P., D. B., I. P. and G. H. supported this project; The manuscript was written through contributions of all authors.
